# Improvement of high-density lipoprotein atheroprotective properties in patients with systemic lupus erythematosus after belimumab treatment

**DOI:** 10.1093/rheumatology/keae192

**Published:** 2024-03-21

**Authors:** Anastasia-Georgia Dedemadi, Christina Gkolfinopoulou, Dimitra Nikoleri, Myrto Nikoloudaki, Hanna Ruhanen, Minna Holopainen, Reijo Käkelä, Georgia Christopoulou, Stavros Bournazos, Pantelis Constantoulakis, Prodromos Sidiropoulos, George Bertsias, Angeliki Chroni

**Affiliations:** Institute of Biosciences and Applications, National Center for Scientific Research “Demokritos”, Agia Paraskevi, Athens, Greece; Department of Chemistry, National and Kapodistrian University of Athens, Zografou, Athens, Greece; Institute of Biosciences and Applications, National Center for Scientific Research “Demokritos”, Agia Paraskevi, Athens, Greece; Laboratory of Rheumatology, Autoimmunity and Inflammation, University of Crete Medical School, Heraklion, Greece; Institute of Molecular Biology and Biotechnology, FORTH, Heraklion, Greece; Laboratory of Rheumatology, Autoimmunity and Inflammation, University of Crete Medical School, Heraklion, Greece; Helsinki University Lipidomics Unit, HiLIPID, Helsinki Institute of Life Science, HiLIFE, and Biocenter Finland, Helsinki, Finland; Molecular and Integrative Biosciences Research Program, Faculty of Biological and Environmental Sciences, University of Helsinki, Helsinki, Finland; Helsinki University Lipidomics Unit, HiLIPID, Helsinki Institute of Life Science, HiLIFE, and Biocenter Finland, Helsinki, Finland; Molecular and Integrative Biosciences Research Program, Faculty of Biological and Environmental Sciences, University of Helsinki, Helsinki, Finland; Helsinki University Lipidomics Unit, HiLIPID, Helsinki Institute of Life Science, HiLIFE, and Biocenter Finland, Helsinki, Finland; Molecular and Integrative Biosciences Research Program, Faculty of Biological and Environmental Sciences, University of Helsinki, Helsinki, Finland; Genotypos-Science Labs, Athens, Greece; Genotypos-Science Labs, Athens, Greece; Genotypos-Science Labs, Athens, Greece; Laboratory of Rheumatology, Autoimmunity and Inflammation, University of Crete Medical School, Heraklion, Greece; Institute of Molecular Biology and Biotechnology, FORTH, Heraklion, Greece; Laboratory of Rheumatology, Autoimmunity and Inflammation, University of Crete Medical School, Heraklion, Greece; Institute of Molecular Biology and Biotechnology, FORTH, Heraklion, Greece; Institute of Biosciences and Applications, National Center for Scientific Research “Demokritos”, Agia Paraskevi, Athens, Greece

**Keywords:** systemic lupus erythematosus, biologicals, high-density lipoprotein, atheroprotection, lipidomic profile

## Abstract

**Objective:**

Chronic inflammatory diseases, like Systemic Lupus Erythematosus (SLE), carry an increased risk for atherosclerosis and cardiovascular events, accompanied by impairment of atheroprotective properties of high-density lipoprotein (HDL). In SLE, serum B cell-activating factor (BAFF), a cytokine implicated in disease progression, has been correlated with subclinical atherosclerosis. We investigated the impact of treatment with belimumab -an anti-BAFF monoclonal antibody- on HDL atheroprotective properties and composition in SLE patients.

**Methods:**

Serum samples were collected from 35 SLE patients with active disease despite conventional therapy, before and after 6-month add-on treatment with belimumab, and 26 matched healthy individuals. We measured cholesterol efflux and antioxidant capacities, paraoxonase-1 (PON1) activity, serum amyloid A1 (SAA1), myeloperoxidase (MPO) and lipid peroxidation product levels of HDL. LC-MS/MS was performed to analyse the HDL lipidome.

**Results:**

Following treatment with belimumab, cholesterol efflux and antioxidant capacities of HDL were significantly increased in SLE patients and restored to levels of control subjects. HDL-associated PON1 activity was also increased, whereas lipid peroxidation products were decreased following treatment. HDL cholesterol efflux and antioxidant capacities correlated negatively with the disease activity. Changes were noted in the HDL lipidome of SLE patients following belimumab treatment, as well as between SLE patients and healthy individuals, and specific changes in lipid species correlated with functional parameters of HDL.

**Conclusions:**

HDL of SLE patients with active disease displays impaired atheroprotective properties accompanied by distinct lipidomic signatures compared with controls. Belimumab treatment may improve the HDL atheroprotective properties and modify the HDL lipidomic signature in SLE patients, thus potentially mitigating atherosclerosis development.

## Graphical abstract

**Graphical Abstract:**
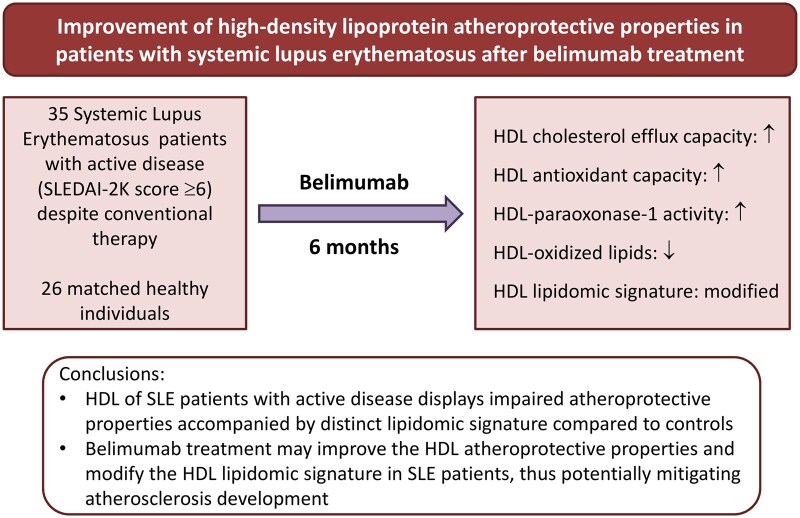

Rheumatology key messages

- HDL atheroprotective properties are improved in patients with systemic lupus erythematosus (SLE) following belimumab treatment.
- Belimumab treatment modifies the HDL lipidomic signature in SLE patients.
- HDL of SLE patients displays impaired atheroprotective properties accompanied by distinct lipidomic signatures compared to controls.

## Introduction

Systemic Lupus Erythematosus (SLE) is a chronic autoimmune disease that exhibits a wide array of systemic effects, involving nearly all organs and tissues [1]. Similar to other chronic inflammatory diseases, SLE is characterized by excessive inflammation and carries a significant risk of cardiovascular disease (CVD). This is thought to be the result of a complex interplay encompassing immune dysregulation and inflammation, higher occurrence of traditional cardiovascular risk factors and accelerated progression of atherosclerosis [2]. Despite advances in the management of the disease, SLE patients still face increased CVD morbidity and mortality as compared with the general population [2].

High-density lipoprotein (HDL) has been suggested to be protective against atherosclerotic CVD through several atheroprotective properties, including cholesterol efflux capacity, antioxidant, anti-inflammatory and immune-regulating activities [3]. A recent meta-analysis showed that higher cholesterol efflux and antioxidant/anti-inflammatory capacities promoted by HDL are linked to a lower risk of CVD and all-cause mortality [4]. HDL particles are heterogeneous lipid/protein complexes that contain apolipoprotein A-I (apoA-I) as well as a variety of other proteins, different lipids and small RNAs [5]. These HDL components participate in the molecule’s pleiotropic functions. Both compositional and functional properties of HDL are modified under pathogenic conditions, including inflammatory diseases [3, 5].

In the context of chronic rheumatic diseases, HDL can lose its atheroprotective functions and even acquire detrimental properties [3, 5]. Previous studies showed that HDL from SLE patients had decreased levels of apoA-I, decreased activity of antioxidant enzyme paraoxonase-1 (PON1) and increased levels of proinflammatory protein serum amyloid A1 (SAA1) and pro-oxidant enzyme myeloperoxidase (MPO) [6, 7]. Furthermore, HDL from SLE patients displayed impaired capacities for cholesterol removal from macrophages, as well as reduced antioxidant and anti-inflammatory actions [6–9]. Chronic inflammation and elevated oxidative stress in SLE [10] may also lead to modifications in the lipid composition of HDL, thereby altering its protective functions. Although several lipidomic analyses have been performed in the plasma of SLE patients showing altered lipid profiles in patients compared with controls [11–14], studies focusing on HDL are missing.

The treatment of SLE includes antimalarials, glucocorticoids and disease-modifying antirheumatic drugs (DMARDs), which induce variable effects on atherosclerosis and CVD risk, yet their effect on HDL functionality is largely unexplored. Antimalarials, such as hydroxychloroquine, and the DMARDs mycophenolate mofetil and methotrexate seem to be protective against atherosclerosis and to improve certain CVD risk factors [2]. On the other hand, azathioprine, calcineurin inhibitors, as well as glucocorticoids, especially at high doses, are associated with increased atherosclerosis and adverse metabolic effects [2].

Belimumab, the first biological agent approved for the treatment of SLE, is a monoclonal antibody that targets B cell activating factor (BAFF)/B lymphocyte stimulator (BLyS) therefore suppressing B cell activation and maturation [15]. In patients with SLE, belimumab may slow the accrual of irreversible organ damage (including from the cardiovascular domain) [16]. However, whether the drug exerts atheroprotective effects remains unknown [2]. BAFF has been implicated in atherosclerosis, but its role is controversial. BAFF-receptor-deficient mouse models of dyslipidaemia displayed a reduction of the atherosclerotic plaque size [17, 18]. By contrast, BAFF overexpression in non-autoimmune mouse models of dyslipidaemia resulted in reduced atherosclerosis and likewise, anti-BAFF antibody treatment increased atherosclerosis [19, 20]. Finally, studies using an atherosclerosis/lupus-prone mouse model showed that anti-BAFF treatment improved atherosclerosis lesions in mice with low plasma cholesterol levels, but worsened the lesions in counterparts with high cholesterol levels [21].

In SLE patients, serum BAFF levels were positively associated with subclinical atherosclerosis [21] and administration of belimumab restored, both *ex vivo* and *in vitro*, the quantitative and qualitative changes of endothelial progenitor cells, whose impairment is associated with increased subclinical atherosclerosis [22]. To this end, there are no subclinical atherosclerosis and cardiovascular event data for patients taking belimumab. However, no abnormal CVD event data have been reported in clinical trials [2].

Herein, we evaluated the effect of belimumab administration on the HDL atheroprotective properties and composition in patients with active SLE despite conventional therapy. The functions and composition of HDL from patients before and after treatment were compared with those of control subjects.

## Materials and methods

### Study design and clinical characteristics

Study participants were recruited by consecutive sampling method from the Department of Rheumatology and Clinical Immunology of the University Hospital of Heraklion, Greece. Thirty-five adult SLE patients who fulfilled the American College of Rheumatology (ACR) revised criteria for the SLE classification [23] were evaluated. Disease activity was measured by the Systemic Lupus Erythematosus Disease Activity Index-2000 (SLEDAI-2K) and organ damage by the Systemic Lupus International Collaborating Clinics Damage Index (SDI). Demographic data, history of cardiovascular risk factors, history of cardiovascular diseases and pharmacological treatment were recorded. Anthropometric parameters were also measured. All patients were started and continued belimumab treatment for 6 months due to active disease and according to the current treatment recommendations [24]. All other treatments (including for comorbidities) had to be stable for the last 12 weeks and throughout the study period. No explicit exclusion criteria were applied in terms of baseline cholesterol levels or intake of lipid-lowering therapies. Twenty-six control subjects who exhibited comparable age, sex, weight and height with SLE patients were recruited. All study participants, patients and controls, were whites. The study was approved by the Ethics Committee of the University Hospital of Heraklion (protocol no. 5944/14–6-2017), and all participants signed informed consent.

Laboratory investigations were performed after overnight fasting as described in Supplementary Data S1, available at *Rheumatology* online.

### Functional and compositional characterization of HDL and genetic analysis

Measurements of cholesterol efflux and antioxidant capacities of HDL, paraoxonase and arylesterase activities of HDL-associated PON1, levels of MPO, SAA1 and lipid peroxidation products in HDL, as well as lipidomic analysis of HDL by LC-MS/MS were performed as described in Supplementary Data S1. Moreover, we screened our sample of SLE patients and controls for the presence of variants in genes with established roles in HDL metabolism and atherosclerosis.

## Results

### Study group characteristics

At baseline, SLE patients had longstanding disease with high disease activity (SLEDAI-2K score ≥6; Table 1). All patients were also receiving treatment with glucocorticoids (58.3%) and/or DMARDs including combinations of hydroxychloroquine (72.2%), methotrexate (33.3%), azathioprine (30.6%), mycophenolate mofetil or leflunomide (8.3% each). The demographic, lifestyle and clinical characteristics of patients and controls are summarized in Table 1. Treatment with belimumab resulted in a significant reduction of the SLEDAI score from (mean ± SD) 7.9 ± 3.3 to 5.3 ± 3.3 (*P* < 0.0001). Accordingly, 19 patients (52.8%) reached a low disease activity state (LLDAS) [25] by 6 months of treatment. Serum total cholesterol, HDL-cholesterol, LDL-cholesterol, triglycerides, HDL-phospholipids, apoA-I and apoB concentrations did not differ statistically either between patients at baseline and following treatment with belimumab, or between patients and controls.

**Table 1.** Clinical characteristics, lipids, and lipoproteins of SLE patients, before and following treatment with belimumab and controls

	SLE patients (n=35)		
	Baseline (T0)	6 months (T6)	Controls (n=26)
Age, yrs	51.2 ± 11.8		44.2 ± 13.1*
Female sex, n (%)	34 (97.1)		26 (100)
Current smokers, n (%)	10 (28.6)	9 (25.7)	5 (19.2)
BMI, kg/m^2^	27.9 ± 5.7	28.0 ± 6.0	25.6 ± 6.2
dsDNA antibody positivity, n (%)^a^	11 (31.4)		
aPL antibodies positivity, n (%)	6 (17.1)		
Disease duration (years)	9.0 ± 8.8		
SLEDAI-2K	7.91 ± 3.25	5.26 ± 3.32**	
SLICC/ACR Damage Index	0.66 ± 0.94	0.69 ± 0.96	
Glucocorticoids use, n (%)	21 (60.0)	18 (51.4)	
Other treatment			
HCQ	26 (74.3)	25 (71.4)	
MMF	3 (8.6)	3 (8.6)	
MTX	12 (34.3)	11 (31.4)	
AZA	11 (31.4)	14 (40.0)	
LEF	3 (8.6)	2 (5.7%)	
RTX	0	0	
CYC	0	0	
CsA	0	0	
ESR, mm/h	25.1 ± 16.7	26.4 ± 19.5	
CRP, mg/dl	0.54 ± 0.65	0.52 ± 0.75	
Hypertension, n (%)	10 (28.6)	10 (28.6)	0
Coronary artery disease (%)	3 (8.6)	3 (8.6)	0
Stroke (%)	1 (2.9)	1 (2.9)	0
Peripheral vascular disease (%)	0	0	0
Type 2 diabetes mellitus, n (%)	3 (8.6)	3 (8.6)	0
Lipid-lowering treatment (%)	7 (20)	7 (20)	0
Total cholesterol, mg/dl	182.6 ± 30.1	180.6 ± 33.1	169.2 ± 30.9
LDL-cholesterol, mg/dl	92.9 ± 27.6	94.7 ± 31.2	89.5 ± 35.7
Triglycerides, mg/dl	149.9 ± 83.3	134.4 ± 73.9	149.9 ± 80.9
apoB, mg/dl	126.9 ± 33.4	123.7 ± 27.9	139.1 ± 38.1
HDL-cholesterol, mg/dl	57.8 ± 36.1	58.6 ± 12.7	55.8 ± 15.5
HDL-phospholipids, mg/dl	112.0 ± 24.3	114.4 ± 20.9	102.3 ± 17.2
apoA-I, mg/dl	156.3 ± 33.3	155.9 ± 31.8	162.0 ± 28.9

Values are mean ± SD unless otherwise indicated.

a Thirty-two out of 35 patients had positive ANA and all patients fulfilled the revised 1997 ACR classification criteria for SLE. The three ANA-negative SLE patients had the following features: patient #1: polyarthritis, photosensitivity, excessive hair loss, malar rash/acute cutaneous lupus, mucosal ulcers, Raynaud’s, skin vasculitis (ACR criteria: 4/11); patient #2: polyarthritis, malar rash, photosensitivity, mucosal ulcers, hair loss, urticaria, lymphopenia <1500/mm^3^ on ≥2 occasions (ACR criteria: 5/11); patient #3: polyarthritis, low C3 and low C4, mucosal ulcers, photosensitivity, acute cutaneous lupus, hair loss, leukopenia <4000 mm/3 on ≥2 occasions (ACR criteria: 4/11).

* *P* < 0.05,

** *P* < 0.0001 compared with baseline.

BMI: body mass index; dsDNA: double-strand DNA; aPL antibodies: antiphospholipid antibodies; SLEDAI-2K: Systemic Lupus Erythematosus Disease Activity Index-2000; SLICC/ACR Damage Index: Systemic Lupus International Collaborating Clinics/American College of Rheumatology Damage Index; HCQ: hydroxychloroquine; MMF: mycophenolate mofetil; MTX: methotrexate; AZA: azathioprine; LEF: leflunomide; RTX: rituximab; CYC: cyclophosphamide; CsA: ciclosporin; ESR: erythrocyte sedimentation rate; CRP: C-reactive protein.

### Atheroprotective properties and composition of HDL from SLE patients before and after treatment with belimumab and from control subjects

**Figure 1.**
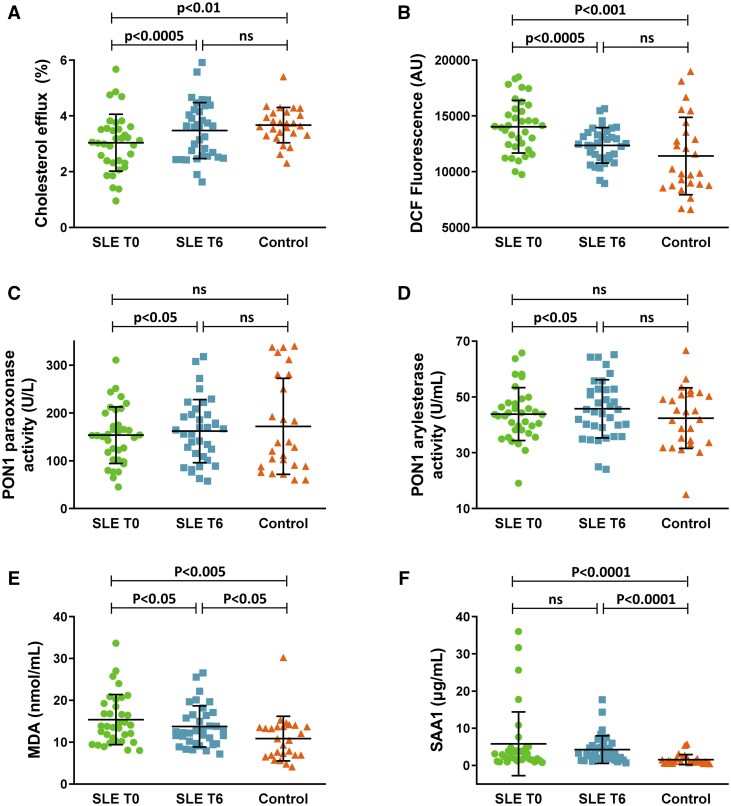
Atheroprotective properties and composition of HDL from SLE patients at baseline (T0) and after 6 months of treatment with belimumab (T6) and from control subjects. (A) % cholesterol efflux from J774 mouse macrophages, treated with chlorophenylthio-cAMP and incubated with HDL particles. (B) Fluorescence intensity resulting from oxidation of DCFH to DCF following incubation of oxLDL with HDL particles. (C) HDL-associated PON1 paraoxonase activity, expressed in U per L. (D) HDL-associated PON1 arylesterase activity, expressed in U per mL. (E) HDL-associated MDA levels, expressed in nmol per mL. (F) HDL-associated SAA1 levels, expressed in μg per mL. Data are shown as mean ± SD. p was calculated by paired *t* test when comparing data between T0 and T6 of treatment and unpaired *t* test when comparing data between patients and controls. ns: not significant

HDL from SLE patients after belimumab treatment had an increased capacity to promote cholesterol efflux as compared with HDL from patients at baseline (Fig. 1A). Notably, HDL from SLE patients after treatment had similar cholesterol efflux capacity as compared with HDL from control subjects, whereas HDL cholesterol efflux capacity from patients at baseline was lower as compared with controls (Fig. 1A).

Measurement of the antioxidant capacity of HDL was performed by the DCF assay, where a decrease in fluorescence signal indicates increased antioxidant potential. In the presence of oxLDL, HDL from SLE patients after belimumab treatment resulted in lower DCF fluorescence signal thus suggesting enhanced antioxidant capacity as compared with HDL before treatment (Fig. 1B). The DCF fluorescence in the presence of HDL from SLE patients post-treatment was at similar levels to the signal obtained with HDL from control subjects (Fig. 1B).

In addition, we examined changes in the activity or levels of proteins (PON1, MPO) of HDL that have been implicated in SLE-related atherosclerosis [6]. Although paraoxonase and arylesterase activities of PON1 were comparable between SLE patients and controls, both were improved in SLE patients following treatment (Fig. 1C and 1D). Measurement of HDL-associated MPO levels in SLE patients showed that the treatment had no significant effect on HDL-MPO levels (Supplementary Fig. S1A, available at *Rheumatology* online). Determination of HDL-associated MDA levels, as a measure of lipid peroxidation [26], showed that MDA levels were higher in SLE patients (before and after treatment) as compared with controls (Fig. 1E). However, the HDL-associated MDA levels decreased in SLE patients following belimumab treatment (Fig. 1E). Pro-inflammatory protein SAA1 levels were also found to be higher in HDL from SLE patients (before and after treatment) as compared with HDL from control subjects (Fig. 1F). Belimumab treatment did not result in any change in HDL-associated SAA1 levels.

**Figure 2.**
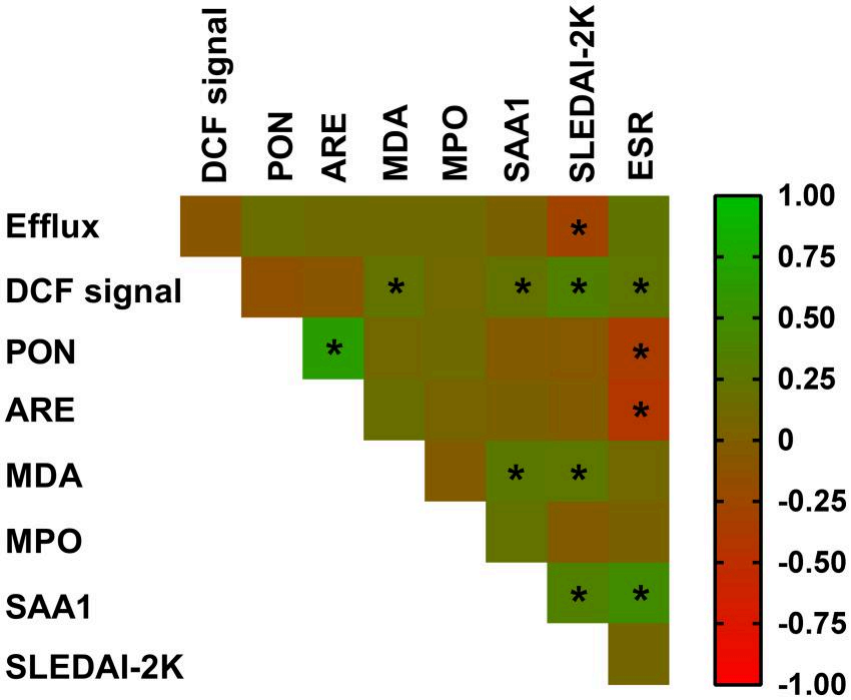
Correlation heatmap demonstrating associations among HDL atheroprotective properties, HDL composition, disease activity and ESR in the combined cohort. Positive and negative correlations are labelled with green and red, respectively; *r* values are colour coded as indicated in the scale. Statistically significant correlations (*P* < 0.05) are indicated with an asterisk. PON: paraoxonase of PON1, ARE: arylesterase activity of PON1

Taken together, these findings suggest that HDL from SLE patients of our cohort, who have a longstanding disease with high disease activity before treatment with belimumab, displays impaired atheroprotective functions and composition as compared with controls. HDL atheroprotective functions and composition though were improved following treatment of SLE patients with belimumab for 6 months. Specific functional and compositional parameters of HDL were inter-correlated and/or correlated with SLE-related parameters, such as the disease activity (SLEDAI-2K) and ESR (Fig. 2). Indeed, DCF signal (indicating reduced HDL antioxidant capacity) was positively correlated with MDA and SAA1 levels in HDL, MDA levels were positively correlated with SAA1 levels and a strong positive correlation was also observed between the two activities of PON1. HDL cholesterol efflux capacity showed a negative correlation with SLEDAI-2K, while DCF signal, MDA levels and SAA1 levels showed a positive correlation with SLEDAI-2K. Furthermore, DCF signal and SAA1 levels were positively correlated with ESR and paraoxonase and arylesterase activities were negatively correlated with ESR.

Comparison of atheroprotective properties of HDL between SLE patients who attained LLDAS and those who did not, showed that belimumab treatment resulted in improvement of HDL cholesterol efflux capacity in both groups (Supplementary Fig. S2A, available at *Rheumatology* online), while the HDL antioxidant capacity was significantly improved only in LLDAS achievers (Supplementary Fig. S2B). The analysis of alterations in the activity and levels of proteins, as well as oxidized lipids of HDL between the LLDAS and non-LLDAS groups, revealed that these parameters exhibited improvement primarily within the former group (Supplementary Fig. S1B and C and Supplementary Fig. S2C–F, available at *Rheumatology* online).

### Lipidome of HDL from SLE patients before and after treatment with belimumab and from control subjects

**Figure 3.**
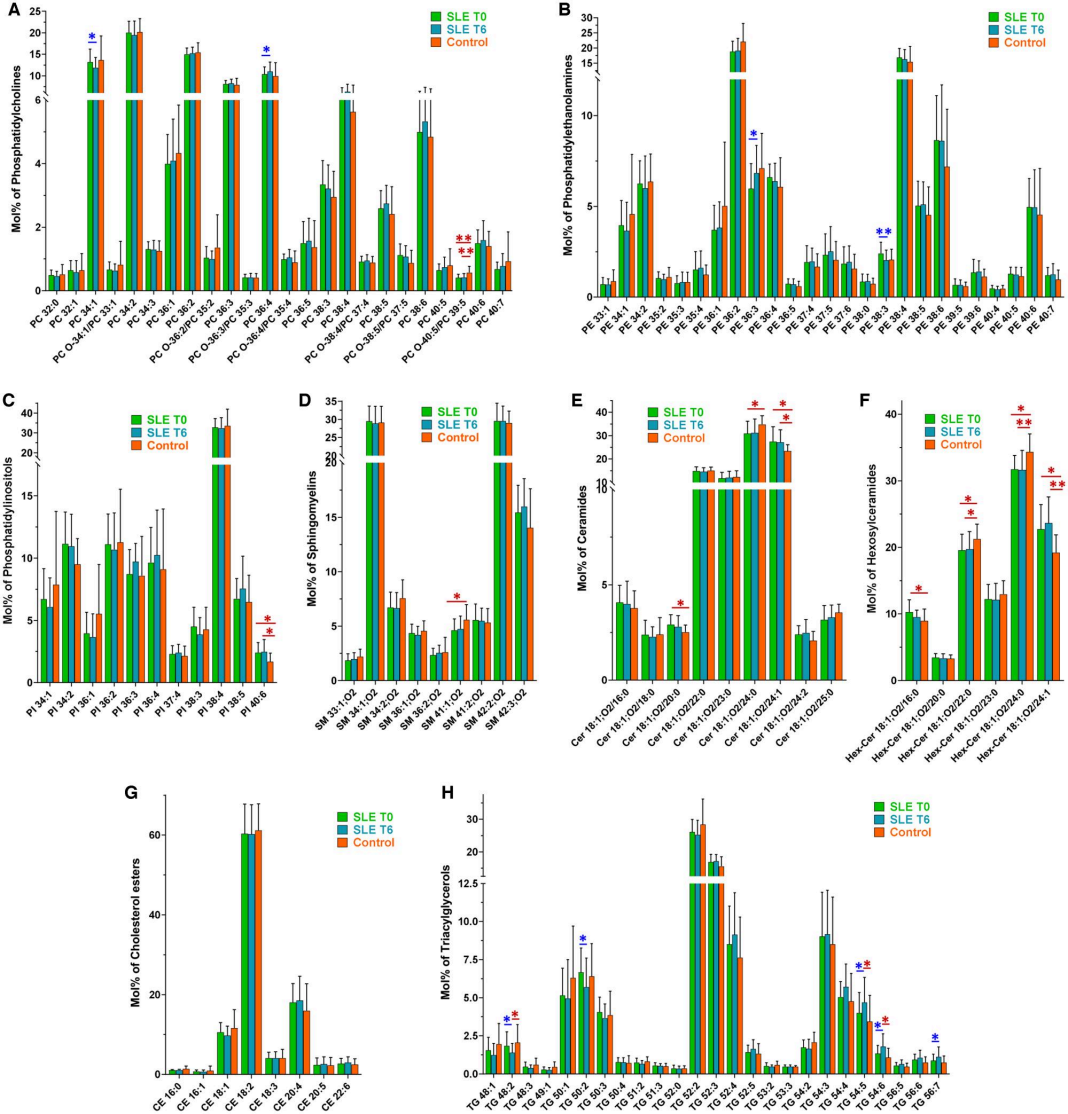
Lipid species of HDL from SLE patients at baseline (T0) and after 6 months of belimumab treatment (T6) and from control subjects. Specifically, panels A-H show the relative species content of (A) phosphatidylcholines [(PC), PC O refers to alkyl species, i.e. an ether bond in sn-1 position instead of an ester bond], (B) phosphatidylethanolamines (PE), (C) phosphatidylinositols (PI), (D) sphingomyelins (SM), (E) ceramides (Cer), (F) hexosylceramides (HexCer), (G) cholesterol esters (CE) and (H) triacylglycerols (TG). Blue asterisks indicate statistically significant differences between T0 and T6 of treatment, calculated by paired *t* test. Red asterisks indicate statistically significant differences between patients (either before or after treatment) and controls, calculated by unpaired *t* test. *P < 0.05; **P < 0.005

An increasing number of studies have indicated that the HDL lipidome can be modified under pathological conditions [27, 28] and several metrics of atheroprotective HDL functionality can be correlated with components of the HDL lipidome [28, 29]. Analysis of several lipid classes (PC, PE, PI, SM, Cer, HexCer, CE and TG) in HDL by LC-MS/MS did not reveal significant differences in their concentration, neither between patients before and after treatment, nor between patients and controls (Supplementary Fig. S3, available at *Rheumatology* online). However, differences were observed in the relative abundances of several lipid species in PC, PE and TG of HDL isolated from patients after treatment as compared with patients before treatment (Fig. 3A, B and H). Additionally, differences in proportions of lipid species in PC, PI, SM, Cer, HexCer and TG were detected between HDL isolated from SLE patients (at baseline or after treatment) and controls (Fig. 3A, C–F, and H).

**Figure 4.**
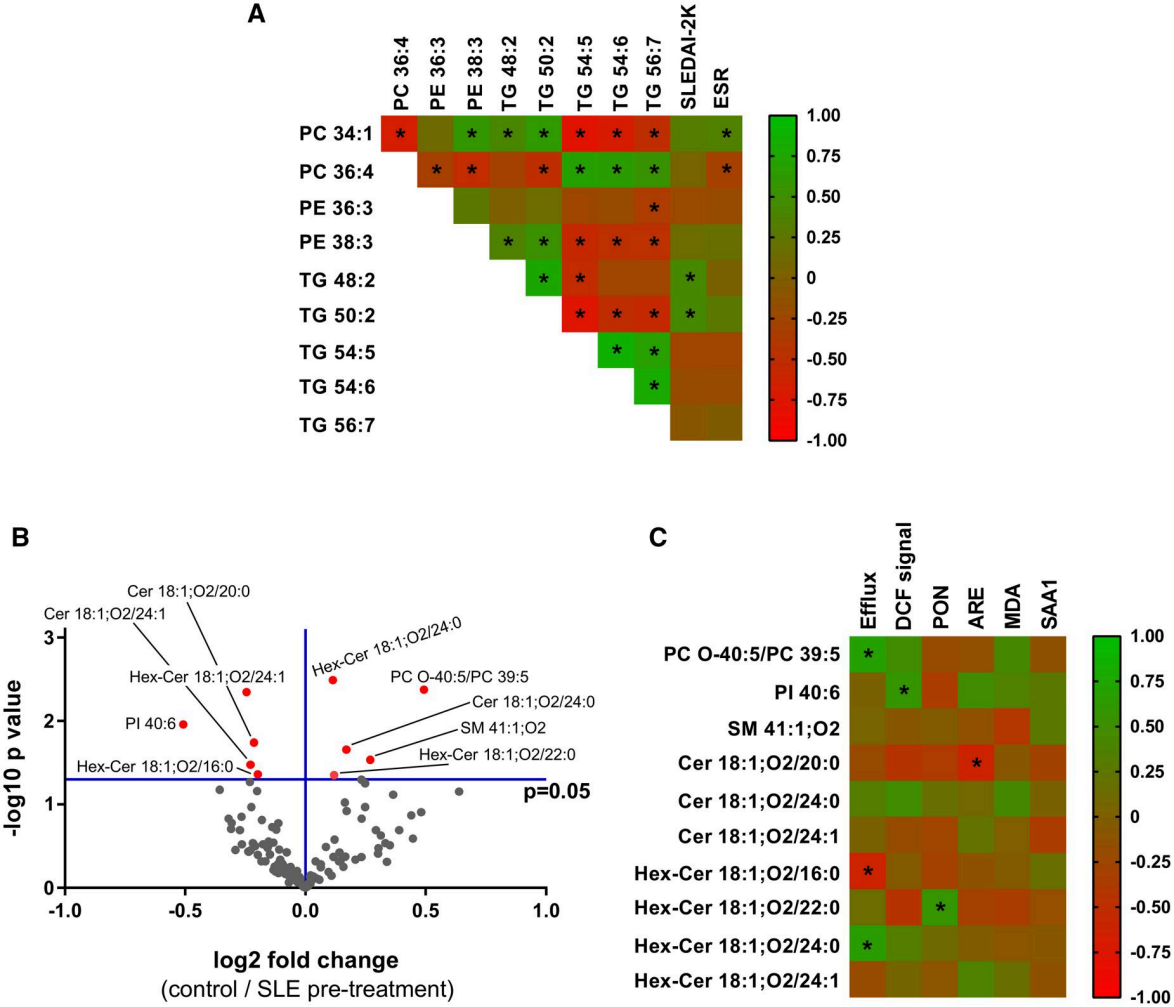
Statistical analyses highlighting associations among HDL lipids, proteins, functions and disease parameters in SLE patients and controls. (A) Correlation heatmap demonstrating associations among the relative concentrations of HDL lipid species that were found significantly changed in SLE patients before and after belimumab treatment, disease activity and ESR. Positive and negative correlations are labelled with green and red, respectively; *r* values are colour coded as indicated in the scale. Statistically significant correlations (*P* < 0.05) are indicated with an asterisk. (B) Volcano plot showing the differences (as log2 fold change) between the relative concentration of each HDL lipid species in control subjects and SLE patients before treatment with belimumab and corresponding p values (as –log10 values). Statistically significant differences (*P* < 0.05) are indicated in red. (C) Correlation heatmap demonstrating associations of relative concentrations of HDL lipid species that were found significantly changed in control subjects as compared with SLE patients before treatment with HDL atheroprotective properties and protein composition. Positive and negative correlations are labelled with green and red, respectively; *r* values are colour coded as indicated in the scale. Statistically significant correlations (*P* < 0.05) are indicated with an asterisk

The majority of HDL PC, PE and TG species that differed between baseline and after treatment were found to be intercorrelated, while specific species correlated with disease activity (SLEDAI-2K) and ESR (Fig. 4A). Specifically, TG 48:2 and TG 50:2 showed a positive correlation with SLEDAI-2K, PC 34:1 a positive correlation with ESR and PC 36:4 a negative correlation with ESR. The characterization of differences in HDL lipidome between SLE patients (before treatment) and controls showed that there were higher levels of PC O-40:5/PC 39:5, SM 41:1; O2, Cer 18:1; O2/24:0, Hex-Cer 18:1; O2/22:0 and Hex-Cer 18:1; O2/24:0 within HDL of controls as compared with SLE patients (Fig. 4B). The levels of PI 40:6, Cer 18:1; O2/20:0, Cer 18:1; O2/24:1, Hex-Cer 18:1; O2/16:0 and Hex-Cer 18:1; O2/24:1 within HDL were lower in controls compared with SLE patients (Fig. 4B). Further analysis showed that functional parameters of HDL correlated with the proportions of some of these lipid species in HDL (Fig. 4C). HDL cholesterol efflux capacity was found to be positively correlated with proportions of PC O-40:5/PC 39:5 and Hex-Cer 18:1; O2/24:0 and negatively correlated with Hex-Cer 18:1; O2/16:0. The DCF signal (indicating reduced HDL antioxidant capacity) correlated positively with PI 40:6. The paraoxonase activity of HDL-PON1 correlated positively Hex-Cer 18:1; O2/22:0 and the arylesterase activity correlated negatively with Cer 18:1; O2/20:0.

### Genetic variation in SLE patients and control subjects

We performed genetic analysis in the cohort of SLE patients and healthy controls to examine whether patients carry specific variants in genes with established roles in HDL metabolism and atherosclerosis (Supplementary Table S1, available at *Rheumatology* online) that could affect HDL atheroprotective functions. Examination of exonic missense variants in genes involved in HDL metabolism did not reveal differences in the relative abundance between SLE and control groups. In addition, we did not observe differences in the relative abundance of various types of variants (synonymous, missense, 3'UTR, 5'UTR, frameshift, intronic etc), in the genes related to HDL metabolism and atherosclerosis that we examined, between SLE and control groups (Supplementary Table S1). These findings indicate that the observed differences in HDL atheroprotective functions between SLE patients and controls in our cohort could not be attributed to genetic variants in genes related to HDL metabolism and atherosclerosis.

Moreover, we examined the relative abundance of variants in genes associated with the occurrence of inflammatory and autoimmune diseases in SLE patients and controls (Supplementary Table S2, available at *Rheumatology* online). The analysis did not reveal any major differences in the relative abundance of variants between the two groups. In addition, HLA haplotype analysis was performed for all SLE and control samples since genetic variation within the major histocompatibility complex has been shown to contribute to substantial risk for SLE [30], as well as for cardiovascular disease [31]. Examination of HLA-A, HLA-B, HLA-C, HLA-DQA1, HLA-DQB1 and HLA-DRB1 alleles (Supplementary Table S3, available at *Rheumatology* online) showed that 8 SLE patients carried the HLA-DQB1*02:02 allele (*vs* 2 control subjects) and 6 patients carried the HLA-DRB1*15:01 allele (*vs* 2 control subjects). HLA-DQB1*02:02 [30] and HLA-DRB1*15:01 [32] alleles have been associated with increased susceptibility to SLE. The current analysis of gene variants associated with inflammatory and autoimmune diseases and of HLA haplotypes does not allow for correlations to be made between genetic variants or HLA alleles and susceptibility to SLE or CVD risk due to the small sample number. However, the findings in our Greek cohort seem to be consistent with previous findings suggesting that an accumulation of polygenic risk factors (involving a large number of common genetic variants with small effect sizes and a few rare variants with large effect sizes) are implicated in disease development [33].

## Discussion

SLE is associated with an increased risk of atherosclerotic cardiovascular disease (CVD) [2]. To our knowledge, the data presented here are the first to describe that treatment with the biological drug belimumab results in improvement of the atheroprotective properties of HDL in SLE patients Our cohort had a relatively high disease activity before treatment with belimumab and the HDL of patients displayed impaired cholesterol efflux and antioxidant capacity, as well as increased levels of SAA1 and lipid peroxidation products as compared with the HDL of controls. This agrees with the results from previous SLE cohorts, characterized by lower disease activity than ours, which showed HDL to have impaired atheroprotective properties and composition compared with healthy individuals [8, 9, 34].

The impaired HDL cholesterol efflux and antioxidant capacities of SLE patients have been linked to subclinical carotid atherosclerosis [34, 35]. The progression of atherosclerosis can be attributed primarily to the systemic inflammatory load that by a complex, incompletely understood, and multifactorial mechanism results in dysregulation of processes (oxidative stress) and function of cells (endothelial, macrophages) involved in the accelerated development of atherosclerosis [2]. Additionally, some non-biologic drugs used for the treatment of SLE, such as azathioprine, calcineurin inhibitors and glucocorticoids, have been associated with increased atherosclerosis and adverse metabolic effects despite improvements in disease activity [2]. Interestingly, a previous study showed that in a small group of SLE patients starting therapy with azathioprine there was no change in the antioxidant function of HDL, while in groups of patients starting therapy with mycophenolate mofetil or hydroxychloroquine, the antioxidant function of HDL was improved [36]. To this end, our findings showing that cholesterol efflux and antioxidant capacity of HDL in SLE patients treated with belimumab for 6 months was restored to the levels of control subjects lay the groundwork for future studies to evaluate the putative salutary effects of belimumab therapy on CVD morbidity and mortality in SLE. The use of other therapies (including glucocorticoids) by the patients remained relatively stable during the 6-month study period, which suggests that the observed differences in HDL properties may be due to belimumab and/or its effect on disease activity.

Accumulating evidence has proposed that HDL functionality may contribute to cardiovascular protection and be relevant as an atherosclerotic CVD biomarker [3]. In agreement with the utility of HDL functionality in CVD risk, a meta-analysis of several studies showed that higher HDL cholesterol efflux and antioxidant capacities were associated with lower CVD risk and lower risk of all-cause mortality [4]. Therefore, the improvement of HDL atheroprotective functions following belimumab treatment might be clinically relevant for CVD risk-reduction strategies in SLE patients.

Previous studies have shown that atheroprotective activities of HDL particles exhibit significant correlations with components of the HDL lipidome [28, 29]. Our analysis of HDL lipid composition in SLE patients and healthy controls also showed that the abundances of specific lipid species are associated with HDL atheroprotective functions and protein components, suggesting that the biological functions of HDL may arise from specific combinations of protein and lipid moieties. Of note, the lipid species Cer 18:1; O2/20:0, Cer 18:1; O2/24:1, Hex-Cer 18:1; O2/16:0 and Hex-Cer 18:1; O2/24:1 that are elevated in the HDL of SLE patients compared with controls were also elevated in the serum of SLE patients [11–14], while SM 41:1; O2 that is reduced in the HDL of SLE patients compared with controls was also reduced in the serum of SLE patients [14]. Whether the HDL lipidome correlates well with the whole-plasma lipidome is largely unexplored given the limited HDL-specific lipidome studies performed either in healthy subjects or in various disease patients. Interestingly, serum Cer 18:1; O2/24:1 and Hex-Cer 18:1; O2/16:0 have been shown to correlate positively with CVD risk [37, 38]. The lipidomic analysis also revealed changes in the abundance of specific lipid species in HDL of SLE patients following belimumab treatment, and four lipid species (PC 34:1, PC 36:4, TG 48:2, TG 50:2) correlated with disease activity and ESR. Two more lipid species, PE 36:3 and PE 38:3, that were increased and decreased, respectively, in HDL of SLE patients after belimumab treatment, displayed a trend to reach the levels of controls following therapy. Notable, the abundance of PE 38:3 in HDL from patients with rheumatoid arthritis was associated with disease activity and CVD risk factors [39]. Overall, this is a pilot study indicating that there are differences in HDL lipidome of SLE patients before and after belimumab treatment, as well as in HDL lipidome of SLE patients compared with controls. Changes in specific HDL lipid species may be related to the presence of SLE as well as disturbances of functional properties of HDL, although the causal links remain to be demonstrated.

Limitations to this study are the small number of subjects and the short observation time. The small number of SLE patients and controls limited the power of statistical analyses, especially for the lipidomic and genomic analyses where small differences were observed. Nonetheless, the observation that changes in the abundance of specific lipid species in the HDL of SLE patients followed a similar trend with changes in the same lipid species in the serum of SLE patients as compared with controls, may indicate that the observed changes in our analysis are valid and specific for SLE.

The short observation time did not allow the effect of treatment and HDL functionality improvement on cardiovascular risk to be determined. Further prospective cohort studies with a larger sample size, longer follow-up and inclusion of a control group treated with conventional drug will be useful to better understand the effects of belimumab on HDL function and composition, as well as on atherosclerosis in SLE.

The current treatment paradigm in SLE aims to achieve low disease activity or remission while also preventing complications, especially irreversible organ damage. In this regard, novel biological agents hold promise based on their established effectiveness in lowering inflammation, reducing flares and tapering glucocorticoids [40]. In agreement with other observational studies [41, 42], a substantial number of patients in our cohort achieved LLDAS following treatment with belimumab. Accordingly, the recent recommendations issued by the European Alliance of Associations for Rheumatology (EULAR) emphasize the treating-to-target approach in SLE, a strategy that can be facilitated with the timely initiation of biological agents [24]. To this end, our study corroborates this concept by demonstrating for the first time, a favourable impact of belimumab on HDL atheroprotective properties, as well as its protein and lipid composition in patients with active SLE. Pending further verification, these findings might account -at least in part- for the effect of belimumab treatment in lowering the risk for organ damage including cardiovascular events [16], therefore, supporting its putative role as a disease-modifying agent.

## Supplementary Material

keae192_Supplementary_Data

## Data Availability

Data will be made available upon reasonable request.
