# Supplementary material for: Improvement of high-density lipoprotein atheroprotective properties in patients with systemic lupus erythematosus after belimumab treatment
Source: Rheumatology (Oxford). 2024 Mar 21;64(2):648–57. doi: 10.1093/rheumatology/keae192 (PMC11781589; doi:10.1093/rheumatology/keae192)
Supplement: keae192_Supplementary_Data [file keae192_supplementary_data.docx]

**Improvement of atheroprotective properties of HDL in patients with systemic lupus erythematosus after belimumab treatment**

Anastasia-Georgia Dedemadi^1,2^, Christina Gkolfinopoulou^1^, Dimitra Nikoleri^3,4^, Myrto Nikoloudaki^3^, Hanna Ruhanen^5,6^, Minna Holopainen^5,6^, Reijo Kakela^5,6^, Georgia Christopoulou^7^, Stavros Bournazos^7^, Pantelis Constantoulakis^7^, Prodromos Sidiropoulos^3,4^, George Bertsias^3,4^ and Angeliki Chroni^1^

^1^Institute of Biosciences and Applications, National Center for Scientific Research “Demokritos”, Agia Paraskevi, Athens, Greece

^2^Department of Chemistry, National and Kapodistrian University of Athens, Zografou, Athens, Greece

^3^Laboratory of Rheumatology, Autoimmunity and Inflammation, University of Crete Medical School, Heraklion, Greece

^4^Institute of Molecular Biology and Biotechnology, FORTH, Heraklion, Greece

^5^Helsinki University Lipidomics Unit, HiLIPID, Helsinki Institute of Life Science, HiLIFE, and Biocenter Finland, Helsinki, Finland

^6^Molecular and Integrative Biosciences Research Program, Faculty of Biological and Environmental Sciences, University of Helsinki, Helsinki, Finland

^7^Genotypos-Science Labs, Athens, Greece

**SUPPLEMENTARY DATA S1. METHODS**

**Biochemical measurements in serum samples**

Laboratory investigations were performed after overnight fasting and included measurements of C-reactive protein (CRP) levels in serum and erythrocyte sedimentation rate (ESR). Total cholesterol, triglycerides, apoA-I and apoB concentrations in serum were also determined using commercially available kits (Cholesterol Liquid-Enzymatic Colorimetric Method, Triglycerides Liquid-Enzymatic Colorimetric Method, Apolipoprotein A-I-Immunoturbidimetric Method and Apolipoprotein B-Immunoturbidimetric Method, Sentinel Diagnostics), according to the manufacturer’s instructions. HDL-cholesterol and HDL-phospholipids were determined in apoB-depleted serum using the Cholesterol Liquid and Phospholipids kits (Sentinel Diagnostics), respectively. LDL cholesterol (LDL-C) was calculated with Friedewald’s formula.

**HDL preparation**

For the analysis of atheroprotective properties and specific protein components of HDL from patients and healthy counterparts, we used apoB-depleted serum, which was isolated by the dextran-Mg^2+^ method as described previously (1,2). For the analysis of HDL lipidome, we used HDL isolated by density gradient ultracentrifugation, following the removal of apoB-containing lipoproteins by the dextran-Mg^2+^ method as described before (3). Briefly, the density of 250 μL apoB-depleted serum (prepared by the dextran-Mg^2+^ method) was adjusted to 1.24 g/mL by the addition of solid KBr. The density-adjusted apoB-depleted serum was transferred in a polycarbonate ultracentrifuge tube and then gently overlaid with 950 μL KBr solution of density 1.21 g/mL. Ultracentrifugation was performed in a Beckman Coulter Optima MAX Tabletop Ultracentrifuge using a fixed-angle rotor MLA-130 for 4 h at 90,000 rpm and 4^o^C. Upon completion of ultracentrifugation, fractions of 100 μL were collected from the top. As judged by SDS-PAGE analysis, the top fractions consist of HDL (d≤1.21 g/mL) and the bottom fractions consist of albumin and other serum proteins. The first six fractions, which contained the majority of pure HDL, were combined and dialyzed against Dulbecco’s phosphate-buffered saline with three buffer changes (the first one overnight) at 4^o^C.

**Cholesterol efflux capacity of HDL**

The cholesterol efflux capacity of HDL was measured as previously described (2). Briefly, J774 mouse macrophages plated in 48-well plates were labeled with 0.25 μCi/mL 4[^14^C]-cholesterol (American Radiolabeled Chemicals, Inc.) in Dulbecco modified Eagle’s medium DMEM (high glucose) supplemented with 0.2% (w/v) bovine serum albumin (BSA) for 24 h. At the end of incubation, the labeling medium was removed and cells were incubated with 0.3 mM 8-(4-chlorophenylthio)-cyclic adenosine monophosphate (cpt-cAMP, Sigma-Aldrich) in DMEM (high glucose) supplemented with 0.2% (w/v) BSA for 24 h. Then, the cpt-cAMP containing medium was removed and cells were incubated with HDL, at a concentration of 10 μg phospholipids /mL, in DMEM (high glucose) supplemented with 0.2% (w/v) BSA for 4 h. Finally, cell supernatants were collected and cleared of cellular debris by brief centrifugation, while cells were lysed in PBS containing 1% (v/v) Triton X-100. The radioactivity in cell supernatant and cell lysate was determined by liquid scintillation counting. The percentage of secreted [^14^C]-cholesterol was calculated by dividing the counts in cell supernatant by the sum of total counts in cell supernatant and cell lysate. All assays were performed in duplicate. To correct for plate-to-plate and day-to-day variations, a reference HDL sample was tested in triplicate on each plate.

**Antioxidant capacity of HDL (DCF assay)**

The antioxidant capacity of HDL was tested in the presence or absence of oxidized LDL (oxLDL) with the DCF assay (1). LDL had been isolated from healthy controls’ serum by ultracentrifugation and exposed to normal atmospheric conditions with gentle mixing at room temperature overnight to generate mildly oxidized LDL (oxLDL). Η_2_DCFDA (2΄,7΄-dichlorodihydrofluorescein diacetate; Thermo Fisher Scientific) was dissolved in methanol at 2.0 mg/mL and incubated at room temperature in the dark for 20 minutes resulting in the release of DCFΗ. Upon interaction with oxidants, non-fluorescent DCFH is oxidized to fluorescent DCF (2΄,7΄-dichlorofluorescein). To compare the HDL antioxidant properties of subjects, we normalized for differences in HDL-C concentration. HDL (final concentration 50 μg cholesterol/mL) was added into a 96-well black plate, in the presence of oxLDL (final concentration 100 μg cholesterol/mL), in a final volume of 100 μL. The plate was incubated at 37^o^C on a rotator (80 rpm) for 1 h in the dark. Then, 10 μL of DCFH solution (0.2 mg/mL) were added to each well and the plate was incubated for an additional 2 h at 37°C with rotation in the dark. Fluorescence was measured in a microplate spectrophotometer (Infinite M200, Tecan Trading AG) at 535 nm, after excitation at 465 nm. An increased fluorescence signal indicates reduced HDL antioxidative activity.

**HDL-associated PON1 activity**

PON1 activity in HDL was determined by measuring both paraoxonase and arylesterase activity of enzyme. Paraoxonase activity assay was performed in a final volume of 250 μL containing 5 μL of HDL, 5.61 mM paraoxon (paraoxon-ethyl, Sigma-Aldrich), 100 mM Tris-HCl and 2 mM CaCl_2_, pH 8.0. The rate of p-nitrophenol formed by the hydrolysis of paraoxon was measured by monitoring the increase in absorbance at 405 nm for 38 minutes at room temperature in a microplate spectrophotometer (Infinite M200, Tecan Trading AG). Paraoxonase activity was expressed as U/L of HDL. One U is the activity that catalyzes the formation of 1 μmol p-nitrophenol per minute.

Arylesterase activity assay was performed in a final volume of 250 μL containing 5 μL of HDL diluted 1:50, 1 mM phenyl acetate (Sigma-Aldrich), 100 mM Tris-HCl and 2 mM CaCl_2_, pH 8.0. The rate of phenol formed by the hydrolysis of phenyl acetate was measured by monitoring the increase in absorbance at 270 nm for 5 minutes at 25^o^C in a microplate spectrophotometer (Spark, Tecan Trading AG). Arylesterase activity was expressed as U/mL of HDL. One U is the activity that catalyzes the formation of 1 μmol phenol per minute.

**HDL-containing MDA levels**

The levels of malondialdehyde (MDA) were measured in HDL by the Thiobarbituric Acid Reactive Substances (TBARS) assay (4) with some modifications. 20 μL of HDL were mixed with 500 μL of 42 mM H_2_SO_4_ aqueous solution and 125 μL of 10% w/v phosphotungstic acid aqueous solution in a microcentrifuge tube and vortexed. Then, the mixture was incubated at room temperature for 5 minutes and centrifuged at 13,000 x g for 3 minutes. The supernatant was discarded and the pellet was resuspended on ice in 200 µL of 0.05 w/v butylated hydroxytoluene (BHT) solution (0.05 w/v in water:methanol 99:1). Standard MDA aqueous solutions at various concentrations (0.2-4 µM) were also prepared by dilution of a 500 μM MDA (Cayman) stock solution. For the assay reaction, 600 μL of TBA (thiobarbityric acid, Cayman) solution (0.335% w/v in water:glacial acetic acid 7:3) were mixed with 200 μL of samples or standards. The mixtures were incubated at 95^o^C for 1 h and then cooled in an ice bath for 10 minutes to stop the reaction. Finally, TBARS were extracted by the addition of 300 μL of 1-butanol and 100 μL of NaCl 5M in each sample and standard and centrifugation at 16,000 x g for 3 minutes. 250 µL of the upper butanol phase, which contains the MDA equivalents (TBARS), were placed in wells of a 96-well plate and fluorescence was measured in a microplate spectrophotometer (Infinite M200, Tecan Trading AG) at 560 nm, after excitation at 530 nm. HDL-associated MDA levels were expressed in nmol per mL.

**HDL-associated SAA1 and MPO levels**

The HDL-associated serum amyloid A1 (SAA1) and myeloperoxidase (MPO) levels were measured using the Human Serum Amyloid A1 DuoSet ELISA kit (Biotechne, R&D Systems) and Human MPO Instant ELISA kit (Invitrogen, Thermo Fisher Scientific), respectively, according to the manufacturer’s instructions.

**Analysis of HDL lipidome**

Lipids were extracted from HDL, isolated from the serum of 20 SLE patients before and after treatment with belimumab and 14 healthy counterparts, by the Folch method (5) and dissolved in chloroform/methanol 1:2 v/v. An internal standard mixture (SPLASH^®^ LIPIDOMIX^®^ Mass Spec Standard, and ceramide d18:1/17:0, Avanti Polar Lipids) was added and the quantification of lipids was carried out using an LC-MS/MS approach. Chromatographic separation was performed using an Agilent 1290 Infinity HPLC system equipped with a Luna Omega C18 100 Å (50 x 2.1 mm, 1.6 µm) column (Phenomenex), and employing an acetonitrile/water/ isopropanol-based gradient solvent system (6) with the flow rate of 0.200 ml/min and 25°C as the column temperature. The column eluent was infused into the electrospray source of an Agilent 6490 Triple Quad LC/MS with iFunnel Technology and spectra were recorded using both positive and negative ionization modes. The lipid classes investigated were phosphatidylcholines (PC), phosphatidylethanolamines (PE), phosphatidylinositols (PI), sphingomyelins (SM), ceramides (Cer), hexosylceramides (HexCer), cholesterol esters (CE) and triacylglycerols (TG). TGs and CEs were detected as [M+NH_4_]^+^ ions and PCs and SMs as [M+H]^+^ ions from MS+ scan. PEs and PIs were detected using head group specific neutral loss scan of 141 amu and precursor ion scan of m/z 241, respectively, and Cers and HexCers using sphingosine 18:1-specific precursor ion scan of m/z 264. Mass spectra were processed using MassHunter Qualitative Navigator software (Agilent) and lipid species were quantified utilizing the internal standards and LIMSA software (7). The lipid species are marked as follows: [lipid class] [sum of acyl chain carbons]:[sum of acyl chain double bonds] (e.g., PC 34:2). Based on inspection of individual ion chromatograms, the PC species annotated with O- were for the most part ether (alkyl-acyl) species with a minor diacyl species component having uneven carbon number, which was also included in the abbreviation. The data are described as concentration (pmol/µL) of lipid classes and as molar percentages (mol%) for each individual species relative to its lipid class.

**Genetic analysis**

Whole genomic DNA was extracted from peripheral blood using standard protocols (QIAamp DNA Mini kit, QIAGEN) and was used for whole-exome sequencing (WES) analysis. Specifically, the exons, adjacent intronic regions (±10 nucleotides), selected regulatory and distal intronic sequences of 21,285 human genome genes were sequenced. For this purpose, a library was constructed with a selection of target regions following hybridization (Twist Human Core Exome EF Multiplex Complete kit, TWIST Bioscience), which was sequenced on a NextSeq500 platform (Illumina). Bioinformatic analysis was performed with validated algorithms using the Sophia DDM analysis system (Sophia Genetics). Overall, 95.5% of the target regions were sequenced to an average depth of 50X. Sequences were mapped to the human reference genome GRCh37/hg19. The nomenclature of the alleles detected follows the latest version of the Human Genome Variation Society guidelines. The selection of genes for the evaluation of the findings was based on specific existing associations of genes with the occurrence of inflammatory diseases and premature atherosclerosis, following virtual panel generation as determined by the Human Phenotype Ontology database (via the Sophia DDM platform), other gene association databases (PanelApp) with the phenotype under investigation and recent literature. For the evaluation and classification of the findings, data from at least the following databases (indicatively) were taken into account: ExAC (r0.3.1), G1000 (v5.20130502), dbSNP (v154), GnomAD (r2.1), ClinVar (v2020817, www.ncbi.nlm.nih.gov/clinvar/), LOVD (www. lovd.nl/), Varsome Data Aggregator (<https://varsome.com/>) as well as from literature and in silico analysis, while the recommendations/guidelines of the American College of Medical Genetics (ACMG-2015 guidelines) were followed.

**Human leukocyte antigen (HLA) haplotype analysis**

For the analysis and typing of HLA in SLE patients’ and controls’ samples, data obtained from the sequencing and bioinformatics analysis by the Sophia DDM system were used. These data were analyzed with the Omixon HLA Explore software (Omixon Biocomputing Ltd.). This program uses a Statistical Genotyping algorithm that aligns the identified sequences with all exonic sequences defined in the IMGT/HLA database. The allele pairs with the highest alignment score are those that are reported. Alleles HLA-A, HLA-B, HLA-C, HLA-DQA1, HLA-DQB1 and HLA-DRB1 were analyzed.

**Statistical analysis**

Statistical analyses were performed using the Prism software (GraphPad Software). Data are presented as percentages for categorical variables and as mean ± standard deviation (SD) for continuous variables. Differences in parameters between pre- and post-treatment periods were compared by paired t-test, while differences between each SLE group (at baseline or after treatment) and the healthy control group were compared by unpaired t-test. Differences in categorical variables between patients and controls were analyzed for significance by Fisher’s exact test. Correlations between variables were evaluated using Pearson’s correlation coefficient for parametric data and Spearman’s correlation coefficient for non-parametric data, following the examination of the normality of distribution for each studied parameter by the Shapiro-Wilk test. For the lipidomics data analysis we employed Bonferroni correction to adjust for multiple testing. p values <0.05 were considered significant.

**References**

1. Tziomalos, K., Katrini, K., Papagianni, M., Christou, K., Gkolfinopoulou, C., Angelopoulou, S. M., Sofogianni, A., Savopoulos, C., et al. (2019) Impaired antioxidative activity of high-density lipoprotein is associated with more severe acute ischemic stroke. *Metabolism* **98**, 49-52

2. Papagiannis, A., Gkolfinopoulou, C., Tziomalos, K., Dedemadi, A. G., Polychronopoulos, G., Milonas, D., Savopoulos, C., Hatzitolios, A. I., et al. (2023) HDL cholesterol efflux capacity and phospholipid content are associated with the severity of acute ischemic stroke and predict its outcome. *Clin. Chim. Acta* **540**, 117229

3. Collins, H. M., Sulpizio, A. C., and Adelman, S. J. (2012) An Improved Method for the Isolation of HDL from Human Serum. *Arterioscler. Thromb. Vasc. Biol.* **32, suppl_1**, A288

4. Yagi, K. (1998) Simple assay for the level of total lipid peroxides in serum or plasma. *Methods Mol. Biol*. **108**, 101-106

5. Folch, J., Lees, M., and Sloane Stanley, G. H. (1957) A simple method for the isolation and purification of total lipides from animal tissues. *J. Biol. Chem.* **226**, 497-509

6. Breitkopf, S. B., Ricoult, S. J. H., Yuan, M., Xu, Y., Peake, D. A., Manning, B. D., and Asara, J. M. (2017) A relative quantitative positive/negative ion switching method for untargeted lipidomics via high resolution LC-MS/MS from any biological source. *Metabolomics* **13**, 30

7. Haimi, P., Uphoff, A., Hermansson, M., and Somerharju, P. (2006) Software tools for analysis of mass spectrometric lipidome data. *Anal. Chem.* **78**, 8324-8331

**SUPPLEMENTARY FIGURES**

**
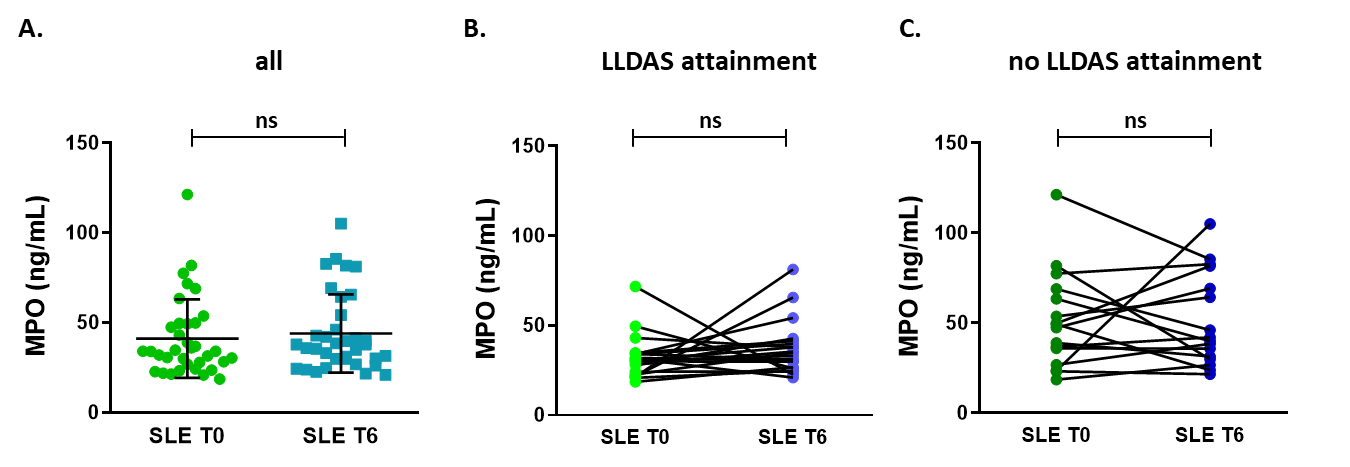
**

**Supplementary Figure S1. HDL-associated MPO levels in SLE patients at baseline (T0) and after 6 months of treatment with belimumab (T6).** Data are shown for the whole group of SLE patients (A), those who attained LLDAS (B) and those who did not attain LLDAS (C). MPO levels are expressed in ng per mL. Data are shown as mean ± SD. ns: not significant difference calculated by paired t test.


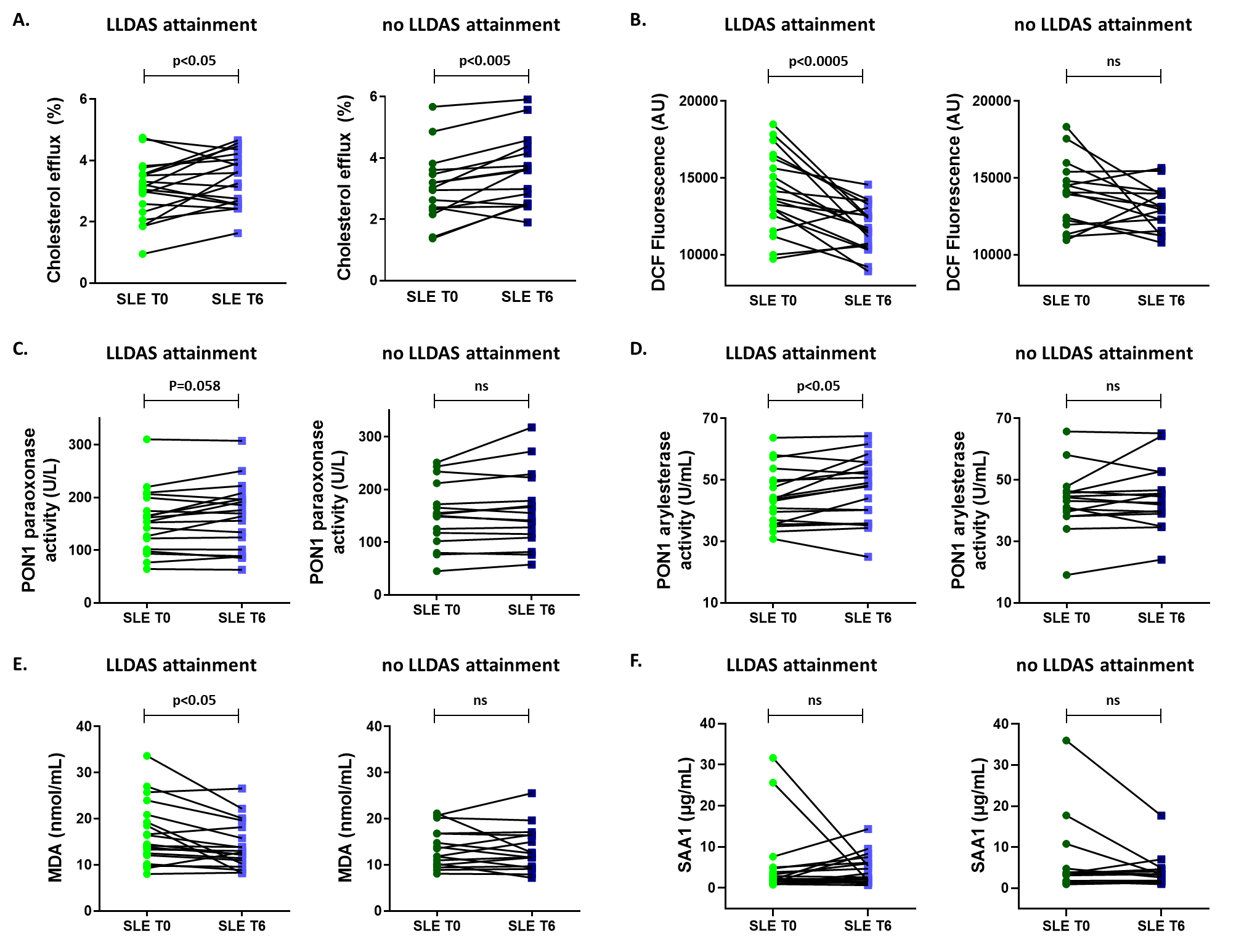


**Supplementary Figure S2. Atheroprotective properties and composition of HDL from SLE patients who attained LLDAS and those who did not attain LLDAS at baseline (T0) and after 6 months of treatment with belimumab (T6).** (A) % cholesterol efflux from J774 mouse macrophages, treated with chlorophenylthio-cAMP and incubated with HDL particles. (B) Fluorescence intensity resulting from oxidation of DCFH to DCF following incubation of oxLDL with HDL particles. (C) HDL-associated PON1 paraoxonase activity, expressed in U per L. (D) HDL-associated PON1 arylesterase activity, expressed in U per mL. (E) HDL-associated MDA levels, expressed in nmol per mL. (F) HDL-associated SAA1 levels, expressed in μg per mL. Data are shown as mean ± SD. p was calculated by paired t test. ns: not significant. AU: arbitrary units.


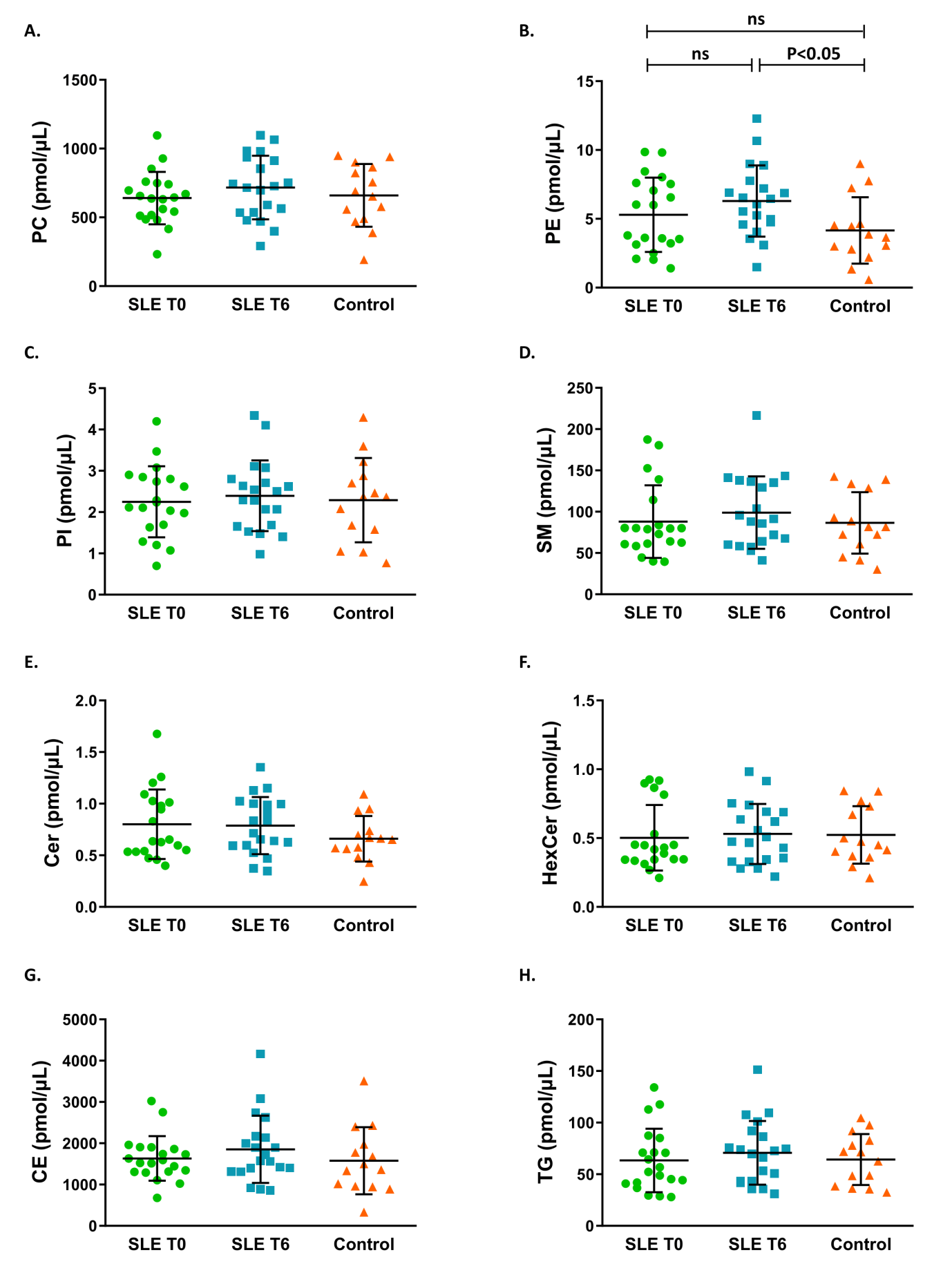


**Supplementary Figure S3. Concentration of lipid classes in HDL from SLE patients at baseline (T0) and after 6 months of treatment with belimumab (T6) and from control subjects.** Specifically, panels A-H show the concentration of (A) phosphatidylcholine (PC), (B) phosphatidylethanolamine (PE), (C) phosphatidylinositol (PI), (D) sphingomyelin (SM), (E) ceramide (Cer), (F) hexosylceramide (HexCer), (G) cholesterol esters (CE) and (H) triacylglycerols (TG) in HDL, expressed in pmol/μL. Data are shown as mean ± SD; Significance of differences between T0 and T6 of treatment was calculated by paired t test, while significance of differences between patients (at baseline or following treatment) and controls was calculated by unpaired t test. ns: not significant

**SUPPLEMENTARY TABLES**

**Supplementary Table S1.** **Variants in genes associated to atherosclerosis identified in SLE patients (n=35) and controls (n=26).**

| **Gene** | **Type** | **Coding Consequence** | **Chr** | **Genome position** | **c.DNA** | **Protein** | **dbSNP** | **SLE**  **n, (%)** | | **control**  **n, (%)** | |
| --- | --- | --- | --- | --- | --- | --- | --- | --- | --- | --- | --- |
| *ABCA1* | SNP | missense | 9 | 107589238 | c.2328G>C | p.(Lys776Asn) | rs138880920 | 1 | (3) | - | |
| *ABCA1* | SNP | 5'UTR | 9 | 107665978 | c.-18G>C |  | rs1800978 | 13 | (37) | 9 | (35) |
| *ABCA1* | SNP | missense | 9 | 107586753 | c.2649A>G | p.(Ile883Met) | rs2066714 | 11 | (31) | 4 | (15) |
| *ABCA1* | SNP | missense | 9 | 107588033 | c.2473G>A | p.(Val825Ile) | rs2066715 | 3 | (9) | 1 | (4) |
| *ABCA1* | SNP | synonymous | 9 | 107568705 | c.4281G>A | p.(Thr1427=) | rs2066716 | 9 | (26) | 5 | (19) |
| *ABCA1* | SNP | missense | 9 | 107589255 | c.2311G>A | p.(Val771Met) | rs2066718 | 2 | (6) | 1 | (4) |
| *ABCA1* | SNP | synonymous | 9 | 107624029 | c.474G>A | p.(Leu158=) | rs2230805 | 19 | (54) | 15 | (58) |
| *ABCA1* | SNP | missense | 9 | 107620867 | c.656G>A | p.(Arg219Lys) | rs2230806 | 20 | (57) | 15 | (58) |
| *ABCA1* | SNP | missense | 9 | 107562804 | c.4760A>G | p.(Lys1587Arg) | rs2230808 | 33 | (94) | 24 | (92) |
| *ABCA1* | SNP | synonymous | 9 | 107602666 | c.948G>A | p.(Gly316=) | rs2246841 | 7 | (20) | 6 | (23) |
| *ABCA1* | SNP | synonymous | 9 | 107602678 | c.936C>T | p.(Pro312=) | rs2274873 | 7 | (20) | 8 | (31) |
| *ABCA1* | SNP | synonymous | 9 | 107591272 | c.2040C>A | p.(Ile680=) | rs2853579 | 10 | (29) | 3 | (12) |
| *ABCA1* | SNP | missense | 9 | 107579632 | c.3516G>C | p.(Glu1172Asp) | rs33918808 | 3 | (9) | 3 | (12) |
| *ABCA1* | SNP | synonymous | 9 | 107578529 | c.3633A>G | p.(Glu1211=) | rs34788556 | 1 | (3) | - | |
| *ABCA1* | SNP | synonymous | 9 | 107581949 | c.3159T>G | p.(Val1053=) | rs35871586 | - | | 1 | (4) |
| *ABCA1* | SNP | missense | 9 | 107558696 | c.5131G>A | p.(Val1711Ile) | rs377600690 | 1 | (3) | - | |
| *ABCA1* | SNP | missense | 9 | 107583705 | c.2911A>G | p.(Ile971Val) | rs57204313 | - | | 1 | (4) |
| *ABCA1* | SNP | intronic | 9 | 107618298 | c.720+2505C>T |  | rs72732679 | 1 | (3) | - | |
| *ABCA1* | SNP | missense | 9 | 107581153 | c.3253A>G | p.(Ile1085Val) | rs760854052 | 1 | (3) | - | |
| *ABCA1* | SNP | synonymous | 9 | 107550222 | c.6183C>T | p.(Gly2061=) | rs9282537 | - | | 6 | (23) |
| *ABCA1* | SNP | missense | 9 | 107599376 | c.1196T>C | p.(Val399Ala) | rs9282543 | 1 | (3) | 1 | (4) |
| *ANGPTL3* | INDEL | nonsense | 1 | 63063667 | c.439_442del | p.(Asn147*) | rs398122987 | 1 | (3) | - | |
| *ANGPTL3* | SNP | missense | 1 | 63069856 | c.1148C>T | p.(Thr383Ile) | rs767910330 | 1 | (3) | - | |
| *ANGPTL4* | SNP | missense | 19 | 8436164 | c.797C>T | p.(Thr266Met) | rs1044250 | 17 | (49) | 13 | (50) |
| *ANGPTL4* | SNP | synonymous | 19 | 8438716 | c.1167G>A | p.(Pro389=) | rs11672433 | 10 | (29) | 10 | (38) |
| *ANGPTL4* | SNP | missense | 19 | 8429323 | c.118G>A | p.(Glu40Lys) | rs116843064 | - | | 3 | (12) |
| *ANGPTL4* | SNP | synonymous | 19 | 8429466 | c.261C>G | p.(Thr87=) | rs774028182 | 1 | (3) | - | |
| *APOA2* | INDEL | intronic | 1 | 161192845 | c.53-11_53-6del |  | rs17244502 | 27 | (77) | 14 | (54) |
| *APOA2* | SNP | intronic | 1 | 161192316 | c.186-4C>T |  | rs6413453 | 7 | (20) | 6 | (23) |
| *APOA4* | SNP | missense | 11 | 116692293 | c.481G>T | p.(Ala161Ser) | rs12721043 | 1 | (3) | - | |
| *APOA4* | SNP | missense | 11 | 116692393 | c.381C>A | p.(Asn127Lys) | rs147610191 | 1 | (3) | 2 | (8) |
| *APOA4* | SNP | synonymous | 11 | 116692324 | c.450C>T | p.(Ala150=) | rs2234668 | 4 | (11) | 5 | (19) |
| *APOA4* | SNP | synonymous | 11 | 116693464 | c.87G>A | p.(Thr29=) | rs5092 | 34 | (97) | 26 | (100) |
| *APOA4* | SNP | synonymous | 11 | 116692552 | c.222T>C | p.(Gly74=) | rs5103 | - | | 1 | (4) |
| *APOA4* | SNP | missense | 11 | 116692334 | c.440G>A | p.(Ser147Asn) | rs5104 | 34 | (97) | 26 | (100) |
| *APOA4* | SNP | missense | 11 | 116691634 | c.1140G>T | p.(Gln380His) | rs5110 | 3 | (9) | 2 | (8) |
| *APOA4* | SNP | missense | 11 | 116691675 | c.1099A>T | p.(Thr367Ser) | rs675 | 12 | (34) | 8 | (31) |
| *APOB* | SNP | missense | 2 | 21233187 | c.6553A>G | p.(Ile2185Val) |  | 1 | (3) | - | |
| *APOB* | SNP | missense | 2 | 21229446 | c.10294C>G | p.(Gln3432Glu) | rs1042023 | 2 | (6) | - | |
| *APOB* | SNP | missense | 2 | 21225753 | c.12541G>A | p.(Glu4181Lys) | rs1042031 | 8 | (23) | 7 | (27) |
| *APOB* | SNP | missense | 2 | 21225281 | c.13013G>A | p.(Ser4338Asn) | rs1042034 | 34 | (97) | 22 | (85) |
| *APOB* | SNP | missense | 2 | 21249716 | c.2188G>A | p.(Val730Ile) | rs12691202 | 1 | (3) | - | |
| *APOB* | SNP | missense | 2 | 21238413 | c.3337G>C | p.(Asp1113His) | rs12713844 | 2 | (6) | - | |
| *APOB* | SNP | missense | 2 | 21252534 | c.1594C>T | p.(Arg532Trp) | rs13306194 | - | | 1 | (4) |
| *APOB* | SNP | missense | 2 | 21263900 | c.293C>T | p.(Thr98Ile) | rs1367117 | 17 | (49) | 11 | (42) |
| *APOB* | SNP | synonymous | 2 | 21238324 | c.3426G>A | p.(Ser1142=) | rs142448733 | - | | 1 | (4) |
| *APOB* | SNP | missense | 2 | 21228386 | c.11354C>T | p.(Thr3785Ile) | rs143710616 | - | | 1 | (4) |
| *APOB* | SNP | missense | 2 | 21246416 | c.2585T>C | p.(Val862Ala) | rs145142090 | - | | 1 | (4) |
| *APOB* | SNP | missense | 2 | 21232125 | c.7615G>A | p.(Val2539Ile) | rs148170480 | - | | 1 | (4) |
| *APOB* | SNP | missense | 2 | 21234056 | c.5684T>C | p.(Val1895Ala) | rs149162499 | 1 | (3) | - | |
| *APOB* | SNP | synonymous | 2 | 21235291 | c.4449A>G | p.(Glu1483=) | rs151018874 | 1 | (3) | - | |
| *APOB* | SNP | synonymous | 2 | 21242741 | c.2853G>A | p.(Glu951=) | rs151193347 | 1 | (3) | - | |
| *APOB* | INDEL | inframe_9 | 2 | 21266774 | c.35_43del | p.(Leu12_Leu14del) | rs17240441 | 15 | (43) | 11 | (42) |
| *APOB* | SNP | synonymous | 2 | 21229609 | c.10131G>A | p.(Leu3377=) | rs1799812 | 1 | (3) | 3 | (12) |
| *APOB* | SNP | missense | 2 | 21224853 | c.13441G>A | p.(Ala4481Thr) | rs1801695 | 6 | (17) | 2 | (8) |
| *APOB* | SNP | missense | 2 | 21233999 | c.5741A>G | p.(Asn1914Ser) | rs1801699 | 1 | (3) | 1 | (4) |
| *APOB* | SNP | synonymous | 2 | 21245813 | c.2706C>T | p.(Asn902=) | rs1801700 | 1 | (3) | - | |
| *APOB* | SNP | missense | 2 | 21228827 | c.10913G>A | p.(Arg3638Gln) | rs1801701 | 6 | (17) | 2 | (8) |
| *APOB* | SNP | missense | 2 | 21225485 | c.12809G>C | p.(Arg4270Thr) | rs1801702 | 5 | (14) | - | |
| *APOB* | SNP | missense | 2 | 21225912 | c.12382G>A | p.(Val4128Met) | rs1801703 | 1 | (3) | 3 | (12) |
| *APOB* | SNP | missense | 2 | 21232803 | c.6936_  6937delinsTG | p.(Ile2313Val) | rs386643884 | 26 | (74) | 16 | (62) |
| *APOB* | SNP | intronic | 2 | 21249840 | c.2068-4T>A |  | rs41291161 | 3 | (9) | - | |
| *APOB* | SNP | missense | 2 | 21233972 | c.5768A>G | p.(His1923Arg) | rs533617 | - | | 2 | (8) |
| *APOB* | SNP | missense | 2 | 21235475 | c.4265A>G | p.(Tyr1422Cys) | rs568413 | 35 | (100) | 26 | (100) |
| *APOB* | SNP | synonymous | 2 | 21233350 | c.6390T>C | p.(Asn2130=) | rs570798466 | - | | 2 | (8) |
| *APOB* | SNP | missense | 2 | 21232803 | c.6937A>G | p.(Ile2313Val) | rs584542 | 26 | (74) | 23 | (88) |
| *APOB* | SNP | missense | 2 | 21225500 | c.12794T>C | p.(Val4265Ala) | rs61743502 | 1 | (3) | 1 | (4) |
| *APOB* | SNP | synonymous | 2 | 21231592 | c.8148C>T | p.(Ile2716=) | rs6413458 | 5 | (14) | - | |
| *APOB* | SNP | missense | 2 | 21231524 | c.8216C>T | p.(Pro2739Leu) | rs676210 | 13 | (37)* | 17 | (65) |
| *APOB* | SNP | missense | 2 | 21250914 | c.1853C>T | p.(Ala618Val) | rs679899 | 21 | (60) | 23 | (88) |
| *APOB* | SNP | synonymous | 2 | 21232195 | c.7545C>T | p.(Thr2515=) | rs693 | 26 | (74) | 16 | (62) |
| *APOB* | SNP | synonymous | 2 | 21232128 | c.7612C>T | p.(Leu2538=) | rs72653093 | 2 | (6) | 2 | (8) |
| *APOB* | SNP | missense | 2 | 21231278 | c.8462C>T | p.(Pro2821Leu) | rs72653095 | 1 | (3) | - | |
| *APOB* | SNP | missense | 2 | 21230828 | c.8912A>C | p.(Asn2971Thr) | rs72653098 | - | | 1 | (4) |
| *APOB* | SNP | missense | 2 | 21227545 | c.11791T>G | p.(Leu3931Val) | rs72654415 | 1 | (3) | - | |
| *APOB* | SNP | missense | 2 | 21225354 | c.12940A>G | p.(Ile4314Val) | rs72654423 | 1 | (3) | 1 | (4) |
| *APOB* | SNP | missense | 2 | 21235202 | c.4538G>A | p.(Arg1513Gln) | rs780817600 | 1 | (3) | - | |
| *APOB* | SNP | missense | 2 | 21230768 | c.8972A>G | p.(Gln2991Arg) |  | - | | 1 | (4) |
| *APOC3* | SNP | splice_donor_+1 | 11 | 116701354 | c.55+1G>A | p.(?) | rs138326449 | 1 | (3) | - | |
| *APOC3* | SNP | synonymous | 11 | 116701535 | c.102T>C | p.(Gly34=) | rs4520 | 32 | (91) | 22 | (85) |
| *APOC3* | SNP | nonsense | 11 | 116701353 | c.55C>T | p.(Arg19*) | rs76353203 | 1 | (3) | - | |
| *APOC3* | SNP | missense | 11 | 116701549 | c.116C>A | p.(Ala39Asp) | rs773670132 | 1 | (3) | - | |
| *APOE* | SNP | missense | 19 | 45411941 | c.388T>C | p.(Cys130Arg) | rs429358 | 10 | (29) | 6 | (23) |
| *APOE* | SNP | missense | 19 | 45409167 | c.42C>G | p.(Asn14Lys) | rs440446 | 29 | (83) | 20 | (77) |
| *APOE* | SNP | missense | 19 | 45412079 | c.526C>T | p.(Arg176Cys) | rs7412 | 3 | (9) | 1 | (4) |
| *APOE* | SNP | missense | 19 | 45411110 | c.137T>C | p.(Leu46Pro) | rs769452 | - | | 1 | (4) |
| *CETP* | SNP | intronic | 16 | 57005479 | c.658+186C>A |  | rs1532624 | 3 | (9) | 2 | (8) |
| *CETP* | SNP | missense | 16 | 57017319 | c.1403G>A | p.(Arg468Gln) | rs1800777 | 2 | (6) | - | |
| *CETP* | SNP | missense | 16 | 57005908 | c.663C>A | p.(Ser221Arg) | rs201438792 | 1 | (3) | - | |
| *CETP* | SNP | missense | 16 | 57015091 | c.1168G>C | p.(Ala390Pro) | rs5880 | 4 | (11) | 1 | (4) |
| *CETP* | SNP | missense | 16 | 57016092 | c.1264G>A | p.(Val422Ile) | rs5882 | 29 | (83) | 23 | (88) |
| *CETP* | SNP | synonymous | 16 | 57007353 | c.861C>T | p.(Phe287=) | rs5883 | 3 | (9) | 2 | (8) |
| *CETP* | SNP | synonymous | 16 | 56995957 | c.66C>A | p.(Thr22=) | rs5884 | 1 | (3) | - | |
| *FADS1* | SNP | intronic | 11 | 61582640 | c.375+1387C>T |  | rs16851310 | - | | 1 | (4) |
| *FADS1* | SNP | intronic | 11 | 61582708 | c.375+1319A>G |  | rs174561 | 4 | (11) | 1 | (4) |
| *GALNT2* | SNP | synonymous | 1 | 230384970 | c.858G>A | p.(Thr286=) | rs1923950 | 6 | (17) | 6 | (23) |
| *GALNT2* | SNP | missense | 1 | 230415148 | c.1660G>A | p.(Val554Met) | rs2273970 | 3 | (9) | - | |
| *GALNT2* | SNP | synonymous | 1 | 230372117 | c.492G>A | p.(Pro164=) | rs34897003 | - | | 1 | (4) |
| *GALNT2* | SNP | synonymous | 1 | 230384937 | c.825T>C | p.(Asp275=) | rs3748006 | 4 | (11) | - | |
| *GALNT2* | SNP | intronic | 1 | 230251823 | c.126+48670G>A |  | rs4846833 | 33 | (94) | 20 | (77) |
| *HMGCR* | SNP | intronic | 5 | 74652762 | c.1986+489G>A |  | rs10942735 | 5 | (14) | 7 | (27) |
| *HMGCR* | INDEL | intronic | 5 | 74652754 | c.1986+485_1986+500del |  | rs17244897 | 24 | (69)* | 8 | (31) |
| *HMGCR* | SNP | intronic | 5 | 74652769 | c.1986+496C>T |  | rs192795914 | - | | 1 | (4) |
| *HMGCR* | SNP | 3'UTR | 5 | 74656175 | c.*8G>A |  | rs5909 | 4 | (11) | 7 | (27) |
| *HNF4A* | SNP | synonymous | 20 | 43034783 | c.201C>T | p.(Ala67=) | rs736823 | - | | 1 | (4) |
| *HNF4A* | SNP | intronic | 20 | 43034693 | c.116-5C>T |  | rs745975 | 11 | (31) | 7 | (27) |
| *LCAT* | SNP | synonymous | 16 | 67974017 | c.1113G>A | p.(Thr371=) | rs368689576 | - | | 1 | (4) |
| *LCAT* | SNP | synonymous | 16 | 67976393 | c.621C>G | p.(Gly207=) | rs370602125 | - | | 1 | (4) |
| *LCAT* | SNP | synonymous | 16 | 67973953 | c.1177C>T | p.(Leu393=) | rs5923 | 3 | (9) | 6 | (23) |
| *LCAT* | SNP | missense | 16 | 67974231 | c.899G>A | p.(Arg300His) | rs770469492 | 1 | (3) | - | |
| *LDLR* | SNP | missense | 19 | 11222300 | c.1171G>A | p.(Ala391Thr) | rs11669576 | 3 | (9) | 4 | (15) |
| *LDLR* | SNP | 3'UTR | 19 | 11242044 | c.*52G>A |  | rs14158 | 12 | (34) | 10 | (38) |
| *LDLR* | SNP | 3'UTR | 19 | 11242215 | c.*223G>A |  | rs17243011 | 1 | (3) | 1 | (4) |
| *LDLR* | SNP | synonymous | 19 | 11227554 | c.1725C>T | p.(Leu575=) | rs1799898 | 16 | (46) | 10 | (38) |
| *LDLR* | SNP | synonymous | 19 | 11210912 | c.81C>T | p.(Cys27=) | rs2228671 | 5 | (14) | 4 | (15) |
| *LDLR* | SNP | intronic | 19 | 11221454 | c.1060+7T>C |  | rs2738442 | 2 | (6) | - | |
| *LDLR* | SNP | 3'UTR | 19 | 11242133 | c.*141G>A |  | rs3826810 | - | | 1 | (4) |
| *LDLR* | SNP | missense | 19 | 11233886 | c.2177C>T | p.(Thr726Ile) | rs45508991 | - | | 1 | (4) |
| *LDLR* | SNP | synonymous | 19 | 11230881 | c.1959T>C | p.(Val653=) | rs5925 | 24 | (69) | 16 | (62) |
| *LDLR* | SNP | synonymous | 19 | 11230842 | c.1920C>T | p.(Asn640=) | rs5926 | - | | 1 | (4) |
| *LDLR* | SNP | synonymous | 19 | 11233941 | c.2232A>G | p.(Arg744=) | rs5927 | 22 | (63) | 21 | (81) |
| *LDLR* | SNP | synonymous | 19 | 11226800 | c.1617C>T | p.(Pro539=) | rs5929 | 2 | (6) | 1 | (4) |
| *LDLR* | SNP | synonymous | 19 | 11224265 | c.1413A>G | p.(Arg471=) | rs5930 | 28 | (80) | 22 | (85) |
| *LDLR* | SNP | synonymous | 19 | 11227602 | c.1773C>T | p.(Asn591=) | rs688 | 24 | (69) | 16 | (62) |
| *LDLR* | SNP | intronic | 19 | 11231203 | c.2140+5G>A |  | rs72658867 | 3 | (9) | 1 | (4) |
| *LIPC* | SNP | missense | 15 | 58855748 | c.1214C>T | p.(Thr405Met) | rs113298164 | - | | 1 | (4) |
| *LIPC* | SNP | missense | 15 | 58860956 | c.1430G>A | p.(Arg477His) | rs148828229 | 1 | (3) | - | |
| *LIPC* | SNP | intronic | 15 | 58857378 | c.1388+1456A>G |  | rs17269397 | 29 | (83) | 25 | (96) |
| *LIPC* | SNP | synonymous | 15 | 58853109 | c.1098A>G | p.(Thr366=) | rs3829461 | 35 | (100) | 26 | (100) |
| *LIPC* | SNP | missense | 15 | 58853079 | c.1068C>A | p.(Phe356Leu) | rs3829462 | 35 | (100) | 26 | (100) |
| *LIPC* | SNP | synonymous | 15 | 58840716 | c.996G>A | p.(Pro332=) | rs537537304 | - | | 1 | (4) |
| *LIPC* | SNP | synonymous | 15 | 58860963 | c.1437C>A | p.(Thr479=) | rs6074 | 13 | (37) | 8 | (31) |
| *LIPC* | SNP | missense | 15 | 58833993 | c.283G>A | p.(Val95Met) | rs6078 | 1 | (3) | 2 | (8) |
| *LIPC* | SNP | synonymous | 15 | 58837957 | c.591A>G | p.(Gly197=) | rs6082 | 3 | (9) | - | |
| *LIPC* | SNP | missense | 15 | 58838010 | c.644A>G | p.(Asn215Ser) | rs6083 | 21 | (60) | 13 | (50) |
| *LIPC* | SNP | synonymous | 15 | 58838038 | c.672C>G | p.(Thr224=) | rs6084 | 30 | (86) | 23 | (88) |
| *LIPC* | SNP | synonymous | 15 | 58834741 | c.465G>T | p.(Val155=) | rs690 | 29 | (83) | 25 | (96) |
| *LIPC* | SNP | intronic | 15 | 58825321 | c.89-5211C>T |  | rs72743026 | - | | 1 | (4) |
| *LIPC* | SNP | missense | 15 | 58855753 | c.1219G>A | p.(Asp407Asn) | rs748608339 | 1 | (3) | - | |
| *LIPC* | INDEL | frameshift | 15 | 58838103 | c.738_739dup | p.(Gly247Alafs*12) | rs749932377 | - | | 1 | (4) |
| *LIPG* | SNP | missense | 18 | 47093864 | c.332C>T | p.(Thr111Ile) | rs2000813 | 20 | (57) | 17 | (65) |
| *LIPG* | SNP | 5'UTR | 18 | 47088655 | c.-24T>G |  | rs34474737 | 23 | (66) | 18 | (69) |
| *LIPG* | SNP | intergenic | 18 | 47088146 |  |  | rs537305372 | 1 | (3) | - | |
| *LIPG* | SNP | missense | 18 | 47107884 | c.893C>G | p.(Thr298Ser) | rs61729805 | 1 | (3) | - | |
| *LIPG* | SNP | missense | 18 | 47109955 | c.1187A>G | p.(Asn396Ser) | rs77960347 | 2 | (6) | - | |
| *LPL* | SNP | missense | 8 | 19805828 | c.226T>G | p.(Phe76Val) |  | - | | 1 | (4) |
| *LPL* | SNP | synonymous | 8 | 19809435 | c.405G>A | p.(Val135=) | rs1121923 | 9 | (26) | 3 | (12) |
| *LPL* | SNP | missense | 8 | 19819628 | c.1325T>G | p.(Val442Gly) | rs116403115 | 1 | (3) | - | |
| *LPL* | SNP | synonymous | 8 | 19813362 | c.786G>A | p.(Gln262=) | rs140986245 | 1 | (3) | - | |
| *LPL* | SNP | missense | 8 | 19805708 | c.106G>A | p.(Asp36Asn) | rs1801177 | 5 | (14) | 1 | (4) |
| *LPL* | SNP | synonymous | 8 | 19810826 | c.435G>A | p.(Glu145=) | rs248 | 1 | (3) | 2 | (8) |
| *LPL* | SNP | synonymous | 8 | 19818436 | c.1164C>A | p.(Thr388=) | rs316 | 5 | (14) | 4 | (15) |
| *LPL* | SNP | nonsense | 8 | 19819724 | c.1421C>G | p.(Ser474*) | rs328 | 13 | (37) | 10 | (38) |
| *LPL* | SNP | 3'UTR | 8 | 19822830 | c.*9G>A |  | rs4922115 | 7 | (20) | 7 | (27) |
| *PCSK9* | SNP | missense | 1 | 55505668 | c.158C>T | p.(Ala53Val) | rs11583680 | 7 | (20) | 6 | (23) |
| *PCSK9* | SNP | missense | 1 | 55505647 | c.137G>T | p.(Arg46Leu) | rs11591147 | 1 | (3) | - | |
| *PCSK9* | SNP | splice_donor_+3 | 1 | 55518467 | c.799+3A>G |  | rs2495477 | 20 | (57) | 19 | (73) |
| *PCSK9* | SNP | synonymous | 1 | 55529047 | c.1869C>T | p.(Thr623=) | rs28362285 | - | | 1 | (4) |
| *PCSK9* | SNP | synonymous | 1 | 55505651 | c.141C>T | p.(Ser47=) | rs28385701 | 1 | (3) | 2 | (8) |
| *PCSK9* | SNP | synonymous | 1 | 55518418 | c.753C>T | p.(Arg251=) | rs28385710 | 1 | (3) | 2 | (8) |
| *PCSK9* | INDEL | inframe_3 | 1 | 55505552 | c.63_65dup | p.(Leu23dup) | rs35574083 | 5 | (14) | 2 | (8) |
| *PCSK9* | INDEL | inframe_6 | 1 | 55505552 | c.60_65dup | p.(Leu22_Leu23dup) | rs35574083 | 3 | (9) | 2 | (8) |
| *PCSK9* | INDEL | inframe_3 | 1 | 55505552 | c.63_65del | p.(Leu23del) | rs35574083 | - | | 1 | (4) |
| *PCSK9* | SNP | missense | 1 | 55529187 | c.2009G>A | p.(Gly670Glu) | rs505151 | 35 | (100) | 26 | (100) |
| *PCSK9* | SNP | synonymous | 1 | 55523033 | c.1026A>G | p.(Gln342=) | rs509504 | 35 | (100) | 26 | (100) |
| *PCSK9* | SNP | synonymous | 1 | 55524197 | c.1380A>G | p.(Val460=) | rs540796 | 35 | (100) | 23 | (88) |
| *PCSK9* | SNP | missense | 1 | 55524237 | c.1420G>A | p.(Val474Ile) | rs562556 | 35 | (100) | 23 | (88) |
| *PCSK9* | SNP | synonymous | 1 | 55509542 | c.234C>T | p.(Tyr78=) | rs779758641 | 11 | (31) | - | |
| *PLTP* | SNP | synonymous | 20 | 44533490 | c.891C>T | p.(His297=) | rs143905045 | - | | 1 | (4) |
| *SCARB1* | SNP | missense | 12 | 125263084 | c.1450C>T | p.(Arg484Trp) | rs10396214 | 1 | (3) | - | |
| *SCARB1* | SNP | missense | 12 | 125263093 | c.1441G>A | p.(Gly481Arg) | rs144985120 | 2 | (6) | 1 | (4) |
| *SCARB1* | SNP | missense | 12 | 125348263 | c.4G>A | p.(Gly2Ser) | rs4238001 | 14 | (40) | 5 | (19) |
| *SCARB1* | SNP | synonymous | 12 | 125284748 | c.1050T>C | p.(Ala350=) | rs5888 | 27 | (77) | 20 | (77) |
| *SCARB1* | SNP | missense | 12 | 125299542 | c.403G>A | p.(Val135Ile) | rs5891 | 1 | (3) | - | |
| *SCARB1* | SNP | synonymous | 12 | 125292413 | c.903C>T | p.(Phe301=) | rs5892 | 1 | (3) | - | |

*p<0.05 compared to control group

**Supplementary Table S2.** **Variants in genes associated to inflammation and autoimmunity identified in SLE patients (n=35) and controls (n=26).** Green color indicates SNPs, associated with SLE susceptibility, that show a trend towards increased frequency in SLE patients as compared to controls. Blue color indicates SNPs with statistically significant rates of occurrence between the two groups.

| **Gene** | **Type** | **Coding** Consequence | **Chr** | **Genome position** | **c.DNA** | **Protein** | **dbSNP** | **SLE**  **n, (%)** | | **control**  **n, (%)** | |
| --- | --- | --- | --- | --- | --- | --- | --- | --- | --- | --- | --- |
| *ACP1* | SNP | synonymous | 2 | 272051 | c.132C>T | p.(Ser44=) | rs11553742 | 7 | (20) | 1 | (4) |
| *ACP1* | SNP | missense | 2 | 272203 | c.284C>T | p.(Thr95Ile) | rs11553746 | 18 | (51) | 12 | (46) |
| *ACP1* | SNP | 3'UTR | 2 | 277314 | c.*10G>A |  | rs77927634 | 2 | (6) | 2 | (8) |
| *ACP1* | SNP | missense | 2 | 277003 | c.317A>G | p.(Gln106Arg) | rs79716074 | 18 | (51) | 12 | (46) |
| *CCR5* | SNP | missense | 3 | 46414580 | c.187A>T | p.(Ser63Cys) | rs142829420 | 1 | (3) | - | |
| *CCR5* | SNP | missense | 3 | 46414557 | c.164T>A | p.(Leu55Gln) | rs1799863 | 2 | (6) | 1 | (4) |
| *CCR5* | SNP | 5'UTR | 3 | 46414384 | c.-10G>C |  | rs184985767 | 1 | (3) | 1 | (4) |
| *CCR5* | INDEL | frameshift | 3 | 46414943 | c.554_585del | p.(Ser185Ilefs*32) | rs333 | 2 | (6) | 1 | (4) |
| *CCR5* | SNP | missense | 3 | 46414784 | c.391G>T | p.(Val131Phe) | rs34418657 | 1 | (3) | 1 | (4) |
| *CCR5* | SNP | missense | 3 | 46414573 | c.180G>T | p.(Arg60Ser) | rs1800940 | - | | 2 | (8) |
| *CD40* | SNP | 5'UTR | 20 | 44746982 | c.-1T>C |  | rs1883832 | 33 | (94) | 23 | (88) |
| *CD40* | SNP | intronic | 20 | 44757407 | c.676-114G>A |  | rs3765459 | 16 | (46) | 6 | (23) |
| *CD40* | SNP | missense | 20 | 44755376 | c.595G>C | p.(Gly199Arg) | rs41282788 | 1 | (3) | - | |
| *CD40* | SNP | missense | 20 | 44751363 | c.371C>T | p.(Ser124Leu) | rs11569321 | - | | 1 | (4) |
| *CD40* | SNP | missense | 20 | 44756823 | c.646C>T | p.(Arg216Trp) | rs7273698 | - | | 1 | (4) |
| *CD40* | SNP | 3'UTR | 20 | 44757707 | c.*28C>T |  | rs765500316 | - | | 1 | (4) |
| *CXCL12* | SNP | synonymous | 10 | 44868465 | c.183G>A | p.(Leu61=) | rs12258838 | 3 | (9) | 1 | (4) |
| *CXCL12* | INDEL | 5'UTR | 10 | 44880456 | c.-7_-4del |  | rs76444314 | 3 | (9) | - | |
| *CXCL12* | SNP | intronic | 10 | 44871372 | c.267-2581G>A |  | rs78342136 | 1 | (3) | - | |
| *CXCL12* | SNP | missense | 10 | 44873247 | c.373C>T | p.(Arg125Cys) | rs200184810 | - | | 1 | (4) |
| *ERAP1* | SNP | missense | 5 | 96122210 | c.1723G>A | p.(Asp575Asn) | rs10050860 | 12 | (34) | 7 | (27) |
| *ERAP1* | SNP | intronic | 5 | 96112083 | c.2818+25A>C |  | rs1065407 | 21 | (60) | 17 | (65) |
| *ERAP1* | SNP | missense | 5 | 96121496 | c.1939G>A | p.(Val647Ile) | rs111363347 | 2 | (6) | - | |
| *ERAP1* | SNP | synonymous | 5 | 96117477 | c.2367T>C | p.(Tyr789=) | rs142985224 | 1 | (3) | - | |
| *ERAP1* | SNP | synonymous | 5 | 96116808 | c.2542C>T | p.(Leu848=) | rs17481856 | 4 | (11) | 7 | (27) |
| *ERAP1* | SNP | missense | 5 | 96118866 | c.2174G>A | p.(Arg725Gln) | rs17482078 | 12 | (34) | 7 | (27) |
| *ERAP1* | SNP | missense | 5 | 96129535 | c.1045A>G | p.(Met349Val) | rs2287987 | 12 | (34) | 7 | (27) |
| *ERAP1* | SNP | missense | 5 | 96130836 | c.828A>G | p.(Ile276Met) | rs26618 | 10 | (29) | 10 | (38) |
| *ERAP1* | SNP | missense | 5 | 96139250 | c.380G>C | p.(Arg127Pro) | rs26653 | 30 | (86) | 24 | (92) |
| *ERAP1* | SNP | missense | 5 | 96118852 | c.2188C>G | p.(Gln730Glu) | rs27044 | 31 | (89) | 25 | (96) |
| *ERAP1* | SNP | synonymous | 5 | 96129512 | c.1068T>C | p.(Ala356=) | rs27434 | 33 | (94) | 26 | (100) |
| *ERAP1* | SNP | synonymous | 5 | 96126308 | c.1359T>C | p.(Ser453=) | rs27529 | 29 | (83) | 25 | (96) |
| *ERAP1* | SNP | missense | 5 | 96129543 | c.1037G>A | p.(Gly346Asp) | rs27895 | 5 | (14) | 4 | (15) |
| *ERAP1* | SNP | missense | 5 | 96124330 | c.1583A>G | p.(Lys528Arg) | rs30187 | 29 | (83) | 25 | (96) |
| *ERAP1* | SNP | synonymous | 5 | 96127833 | c.1251C>T | p.(His417=) | rs3213809 | 3 | (9) | 7 | (27) |
| *ERAP1* | SNP | missense | 5 | 96139464 | c.166G>A | p.(Glu56Lys) | rs3734016 | 4 | (11) | 2 | (8) |
| *ERAP1* | SNP | synonymous | 5 | 96121524 | c.1911G>A | p.(Ala637=) | rs469783 | 27 | (77) | 20 | (77) |
| *ERAP1* | SNP | synonymous | 5 | 96117555 | c.2289G>A | p.(Leu763=) | rs61745685 | 8 | (23) | 7 | (27) |
| *ERAP1* | SNP | missense | 5 | 96139595 | c.35C>T | p.(Thr12Ile) | rs72773968 | 4 | (11) | 7 | (27) |
| *ERAP1* | SNP | missense | 5 | 96139107 | c.523A>T | p.(Arg175Trp) | rs141992697 | - | | 1 | (4) |
| *ERAP1* | SNP | missense | 5 | 96127868 | c.1216G>A | p.(Asp406Asn) | rs181668902 | - | | 1 | (4) |
| *ERAP1* | SNP | missense | 5 | 96124373 | c.1540G>A | p.(Gly514Arg) | rs78649652 | - | | 1 | (4) |
| *ERAP2* | INDEL | intronic | 5 | 96232568 | c.1503+80_  1503+81ins100 |  |  | 1 | (3) | - | |
| *ERAP2* | SNP | missense | 5 | 96245342 | c.2228_  2229delinsAT | p.(Gly743Asp) |  | 1 | (3) | - | |
| *ERAP2* | SNP | synonymous | 5 | 96245439 | c.2325C>T | p.(Ser775=) | rs1056893 | 30 | (86) | 20 | (77) |
| *ERAP2* | SNP | missense | 5 | 96215825 | c.436G>A | p.(Glu146Lys) | rs143635500 | 1 | (3) | - | |
| *ERAP2* | INDEL | intronic | 5 | 96232568 | c.1503+3_  1503+102dup |  | rs1581849348 | 3 | (9) | 5 | (19) |
| *ERAP2* | SNP | missense | 5 | 96239258 | c.2006T>A | p.(Leu669Gln) | rs17408150 | 4 | (11) | 6 | (23) |
| *ERAP2* | SNP | synonymous | 5 | 96244719 | c.2067T>C | p.(His689=) | rs17486915 | 4 | (11) | 6 | (23) |
| *ERAP2* | SNP | splice_donor_+3 | 5 | 96235896 | c.1572+3A>G |  | rs2248374 | 32 | (91) | 20 | (77) |
| *ERAP2* | SNP | synonymous | 5 | 96249115 | c.2611C>T | p.(Leu871=) | rs2255546 | 32 | (91) | 20 | (77) |
| *ERAP2* | SNP | synonymous | 5 | 96237326 | c.1689G>A | p.(Gln563=) | rs2287988 | 32 | (91) | 20 | (77) |
| *ERAP2* | SNP | synonymous | 5 | 96232142 | c.1305T>A | p.(Pro435=) | rs2548538 | 32 | (91) | 20 | (77) |
| *ERAP2* | SNP | missense | 5 | 96231000 | c.1176G>T | p.(Lys392Asn) | rs2549782 | 32 | (91) | 20 | (77) |
| *ERAP2* | SNP | synonymous | 5 | 96245343 | c.2229C>T | p.(Gly743=) | rs2549796 | 31 | (89) | 20 | (77) |
| *ERAP2* | SNP | synonymous | 5 | 96215680 | c.291C>T | p.(Ile97=) | rs41506651 | 12 | (34) | 7 | (27) |
| *ERAP2* | SNP | missense | 5 | 96228072 | c.1040C>T | p.(Thr347Met) | rs75263594 | 5 | (14) | - | |
| *ERAP2* | SNP | missense | 5 | 96219561 | c.641C>T | p.(Pro214Leu) | rs3733905 | - | | 1 | (4) |
| *IL19* | SNP | missense | 1 | 207015897 | c.464A>G | p.(Lys155Arg) |  | 1 | (3) | - | |
| *IL19* | SNP | missense | 1 | 207015957 | c.524T>C | p.(Phe175Ser) | rs2243191 | 31 | (89) | 24 | (92) |
| *IL33* | SNP | synonymous | 9 | 6253571 | c.363C>T | p.(Tyr121=) | rs10975519 | 16 | (46) | 16 | (62) |
| *IL6* | SNP | intergenic | 7 | 22766645 | c.-174G>C |  | rs1800795 | 9 | (26) | 7 | (27) |
| *IL6* | SNP | synonymous | 7 | 22771156 | c.603C>T | p.(Phe201=) | rs2069849 | 3 | (9) | 2 | (8) |
| *IL6* | SNP | missense | 7 | 22771038 | c.485A>T | p.(Asp162Val) | rs2069860 | 1 | (3) | 1 | (4) |
| *IRF5* | SNP | 3'UTR | 7 | 128589000 | c.*128T>C |  | rs2070197 | 3 | (9) | - | |
| *IRF5* | SNP | synonymous | 7 | 128588045 | c.1050T>G | p.(Leu350=) | rs2230117 | 1 | (3) | - | |
| *IRF5* | INDEL | inframe_30 | 7 | 128587351 | c.572_601del | p.(Arg191_  Leu200del) | rs60344245 | 25 | (71) | 18 | (69) |
| *LNPEP* | SNP | 5'UTR | 5 | 96271723 | c.-137A>C |  |  | 1 | (3) | - | |
| *LNPEP* | SNP | missense | 5 | 96363459 | c.2887A>G | p.(Ile963Val) | rs11746232 | 7 | (20) | 5 | (19) |
| *LNPEP* | SNP | 5'UTR | 5 | 96271546 | c.-314C>T |  | rs1416255496 | 1 | (3) | - | |
| *LNPEP* | SNP | synonymous | 5 | 96315371 | c.549G>A | p.(Pro183=) | rs147419734 | 1 | (3) | - | |
| *LNPEP* | SNP | 5'UTR | 5 | 96271440 | c.-420A>T |  | rs17550808 | 4 | (11) | 6 | (23) |
| *LNPEP* | SNP | 5'UTR | 5 | 96271804 | c.-56C>T |  | rs185601392 | 5 | (14) | - | |
| *LNPEP* | SNP | missense | 5 | 96350710 | c.2287G>A | p.(Ala763Thr) | rs2303138 | 6 | (17) | 5 | (19) |
| *LNPEP* | SNP | 5'UTR | 5 | 96271638 | c.-222A>G |  | rs2617434 | 21 | (60) | 17 | (65) |
| *LNPEP* | INDEL | intronic | 5 | 96332085 | c.1408-3del |  | rs3836862 | 32 | (91) | 24 | (92) |
| *LNPEP* | SNP | missense | 5 | 96322360 | c.1117G>A | p.(Val373Ile) | rs41276279 | 4 | (11) | 6 | (23) |
| *LNPEP* | SNP | missense | 5 | 96315320 | c.498C>G | p.(Ile166Met) | rs61752351 | 2 | (6) | - | |
| *LNPEP* | SNP | missense | 5 | 96341912 | c.1921C>T | p.(Pro641Ser) | rs72775875 | 1 | (3) | 2 | (8) |
| *LTA* | SNP | missense | 6 | 31540784 | c.179C>A | p.(Thr60Asn) | rs1041981 | 15 | (43) | 11 | (42) |
| *LTA* | SNP | missense | 6 | 31540757 | c.152A>C | p.(His51Pro) | rs2229092 | 6 | (17) | 6 | (23) |
| *LTA* | SNP | missense | 6 | 31540556 | c.37T>C | p.(Cys13Arg) | rs2229094 | 15 | (43) | 14 | (54) |
| *LTA* | SNP | intronic | 6 | 31540313 | c.-10+90A>G |  | rs909253 | 4 | (11) | - | |
| *MSRA* | SNP | 3'UTR | 8 | 10285825 | c.*3T>G |  | rs10903326 | 35 | (100) | 26 | (100) |
| *MSRA* | SNP | intronic | 8 | 10268701 | c.544-16957G>A |  | rs149970225 | 2 | (6) | 1 | (4) |
| *MSRA* | SNP | synonymous | 8 | 10285810 | c.696T>C | p.(Gly232=) | rs3750314 | 5 | (14) | 1 | (4) |
| *MSRA* | SNP | intronic | 8 | 10211175 | c.543+33676C>T |  | rs6601444 | 10 | (29) | 10 | (38) |
| *MSRA* | SNP | synonymous | 8 | 10285789 | c.675C>T | p.(Thr225=) | rs778456266 | - | | 1 | (4) |
| *MTHFR* | SNP | missense | 1 | 11854476 | c.1286A>C | p.(Glu429Ala) | rs1801131 | 24 | (69) | 18 | (69) |
| *MTHFR* | SNP | missense | 1 | 11856378 | c.665C>T | p.(Ala222Val) | rs1801133 | 22 | (63) | 15 | (58) |
| *MTHFR* | SNP | synonymous | 1 | 11854896 | c.1056C>T | p.(Ser352=) | rs2066462 | 2 | (6) | 5 | (19) |
| *MTHFR* | SNP | synonymous | 1 | 11863057 | c.117C>T | p.(Pro39=) | rs2066470 | 1 | (3) | 5 | (19) |
| *MTHFR* | SNP | missense | 1 | 11850927 | c.1781G>A | p.(Arg594Gln) | rs2274976 | 1 | (3) | - | |
| *MTHFR* | SNP | missense | 1 | 11850750 | c.1958C>T | p.(Thr653Met) | rs35737219 | 2 | (6) | 1 | (4) |
| *MTHFR* | SNP | synonymous | 1 | 11854457 | c.1305C>T | p.(Phe435=) | rs4846051 | 35 | (100) | 26 | 100 |
| *MTHFR* | INDEL | missense | 1 | 11854085 | c.1408_  1409 delinsCT | p.(Glu470Leu) | rs886043349 | 1 | (3) | - | |
| *MTHFR* | SNP | missense | 1 | 11854790 | c.1162C>T | p.(Arg388Cys) | rs200138092 | - | | 1 | (4) |
| *MTHFR* | SNP | missense | 1 | 11852411 | c.1556G>T | p.(Arg519Leu) | rs45449298 | - | | 1 | (4) |
| *NFKB1* | SNP | missense | 4 | 103501701 | c.740A>G | p.(Asn247Ser) | rs1369756216 | 1 | (3) | - | |
| *NFKB1* | INDEL | intronic | 4 | 103534740 | c.2749+11dupA |  | rs148268461 | 3 | (9) | 1 | (4) |
| *NFKB1* | SNP | synonymous | 4 | 103514658 | c.1143T>C | p.(Ala381=) | rs1609993 | 34 | (97) | 26 | (100) |
| *NFKB1* | SNP | missense | 4 | 103518700 | c.1519A>G | p.(Met507Val) | rs4648072 | 1 | (3) | - | |
| *NFKB1* | SNP | synonymous | 4 | 103531736 | c.2232A>G | p.(Ala744=) | rs138657975 | - | | 1 | (4) |
| *NFKB1* | SNP | intronic | 4 | 103501689 | c.731-3C>T |  | rs144345189 | - | | 1 | (4) |
| *NFKB1* | SNP | synonymous | 4 | 103527655 | c.1755G>A | p.(Thr585=) | rs4648093 | - | | 1 | (4) |
| *NFKB1* | SNP | 3'UTR | 4 | 103537774 | c.*23G>A |  | rs4648143 | - | | 1 | (4) |
| *NOS1* | SNP | synonymous | 12 | 117685270 | c.2706C>T | p.(His902=) | rs1047735 | 11 | (31) | 14 | (54) |
| *NOS1* | SNP | missense | 12 | 117693817 | c.2557C>T | p.(Pro853Ser) | rs11068428 | 12 | (34) | 14 | (54) |
| *NOS1* | SNP | missense | 12 | 117693822 | c.2552T>G | p.(Phe851Cys) | rs200972861 | 1 | (3) | - | |
| *NOS1* | SNP | missense | 12 | 117725949 | c.1057G>A | p.(Val353Ile) | rs202090735 | 1 | (3) | - | |
| *NOS1* | SNP | synonymous | 12 | 117657991 | c.4059C>A | p.(Val1353=) | rs2293044 | 2 | (6) | 1 | (4) |
| *NOS1* | SNP | synonymous | 12 | 117701714 | c.2202T>C | p.(Ile734=) | rs2293054 | 33 | (94) | 24 | (92) |
| *NOS1* | SNP | synonymous | 12 | 117665330 | c.3522G>A | p.(Leu1174=) | rs34375182 | 1 | (3) | - | |
| *NOS1* | SNP | synonymous | 12 | 117669914 | c.3258C>T | p.(Asp1086=) | rs3741475 | 10 | (29) | 10 | (38) |
| *NOS1* | SNP | missense | 12 | 117724018 | c.1181A>C | p.(Asp394Ala) | rs9658356 | 2 | (6) | - | |
| *NOS1* | SNP | missense | 12 | 117691500 | c.2591G>A | p.(Gly864Asp) | rs9658445 | 1 | (3) | - | |
| *NOS1* | SNP | synonymous | 12 | 117665264 | c.3588T>C | p.(Thr1196=) | rs9658501 | 2 | (6) | 1 | (4) |
| *NOS1* | SNP | synonymous | 12 | 117669842 | c.3330G>A | p.(Thr1110=) | rs77927749 | - | | 1 | (4) |
| *NOS1AP* | SNP | synonymous | 1 | 162335256 | c.1002C>T | p.(Arg334=) | rs348624 | 10 | (29) | 9 | (35) |
| *NOS1AP* | SNP | synonymous | 1 | 162313735 | c.564C>T | p.(Ser188=) | rs3751284 | 16 | (46) | 16 | (62) |
| *NOS1AP* | SNP | 3'UTR | 1 | 162337263 | c.*6G>A |  | rs971583371 | - | | 1 | (4) |
| *NOS2* | SNP | missense | 17 | 26091123 | c.2476C>G | p.(Gln826Glu) |  | 1 | (3) | - | |
| *NOS2* | SNP | synonymous | 17 | 26092631 | c.2358T>C | p.(Gly786=) | rs1060822 | 34 | (97) | 23 | (88) |
| *NOS2* | SNP | synonymous | 17 | 26089867 | c.2757A>G | p.(Thr919=) | rs1060826 | 34 | (97) | 23 | (88) |
| *NOS2* | SNP | synonymous | 17 | 26105932 | c.1155C>T | p.(Asp385=) | rs1137933 | 11 | (31) | 11 | (42) |
| *NOS2* | SNP | synonymous | 17 | 26115949 | c.204A>G | p.(Pro68=) | rs16966563 | 1 | (3) | 1 | (4) |
| *NOS2* | SNP | missense | 17 | 26096597 | c.1823C>T | p.(Ser608Leu) | rs2297518 | 12 | (34) | 8 | (31) |
| *NOS2* | SNP | intergenic | 17 | 26131326 | TAAA//TAAA |  | rs2779251 | 6 | (17)* | 12 | (46) |
| *NOS2* | SNP | synonymous | 17 | 26125743 | c.93C>T | p.(Ala31=) | rs3730014 | 3 | (9) | 2 | (8) |
| *NOS2* | SNP | missense | 17 | 26109102 | c.661C>T | p.(Arg221Trp) | rs3730017 | 1 | (3) | 1 | (4) |
| *NOS2* | SNP | synonymous | 17 | 26089857 | c.2767C>T | p.(Leu923=) | rs746604115 | 1 | (3) | - | |
| *NOS2* | SNP | missense | 17 | 26107904 | c.893A>G | p.(Lys298Arg) | rs200688826 | - | | 1 | (4) |
| *NOS2* | SNP | synonymous | 17 | 26108167 | c.759C>T | p.(Gly253=) | rs780146841 | - | | 1 | (4) |
| *NOS3* | SNP | missense | 7 | 150704245 | c.1993C>G | p.(Arg665Gly) |  | 1 | (3) | - | |
| *NOS3* | SNP | 3'UTR | 7 | 150711263 | c.*6G>A |  | rs151197128 | 1 | (3) | - | |
| *NOS3* | SNP | synonymous | 7 | 150695726 | c.774T>C | p.(Asp258=) | rs1549758 | 34 | (97) | 25 | (96) |
| *NOS3* | SNP | missense | 7 | 150696111 | c.894T>G | p.(Asp298Glu) | rs1799983 | 34 | (97) | 25 | (96) |
| *NOS3* | SNP | synonymous | 7 | 150704250 | c.1998C>G | p.(Ala666=) | rs2566514 | 13 | (37) | 9 | (35) |
| *NOS3* | SNP | intronic | 7 | 150709571 | c.3106+11G>T |  | rs7830 | 17 | (49) | 12 | (46) |
| *NOS3* | SNP | missense | 7 | 150693603 | c.382C>G | p.(Arg128Gly) |  | - | | 1 | (4) |
| *NOS3* | SNP | missense | 7 | 150696076 | c.859G>A | p.(Asp287Asn) | rs149539813 | - | | 1 | (4) |
| *NOS3* | SNP | missense | 7 | 150698349 | c.1264G>A | p.(Ala422Thr) | rs150935488 | - | | 1 | (4) |
| *NOS3* | SNP | intronic | 7 | 150709580 | c.3106+20G>C |  | rs199658738 | - | | 1 | (4) |
| *PON1* | SNP | missense | 7 | 94937446 | c.575A>G | p.(Gln192Arg) | rs662 | 23 | (66)* | 10 | (38) |
| *PON1* | SNP | missense | 7 | 94946084 | c.163T>A | p.(Leu55Met) | rs854560 | 23 | (66) | 16 | (62) |
| *SMAD3* | SNP | 5'UTR | 15 | 67358478 | c.-15G>A |  | rs1061427 | 19 | (54) | 13 | (50) |
| *SMAD3* | SNP | synonymous | 15 | 67457335 | c.309A>G | p.(Leu103=) | rs1065080 | 35 | (100) | 26 | (100) |
| *SMAD3* | SNP | synonymous | 15 | 67473790 | c.870C>T | p.(Ile290=) | rs117185005 | 2 | (6) | 2 | (8) |
| *SMAD3* | SNP | missense | 15 | 67457698 | c.508A>G | p.(Ile170Val) | rs35874463 | 2 | (6) | 2 | (8) |
| *SMAD3* | SNP | intronic | 15 | 67477061 | c.872-4G>A |  | rs776577220 | - | | 1 | (4) |
| *TGFB1* | SNP | 5'UTR | 19 | 41858953 | c.-4C>T |  | rs1309666668 | 1 | (3) | - | |
| *TGFB1* | SNP | missense | 19 | 41858921 | c.29C>T | p.(Pro10Leu) | rs1800470 | 27 | (77) | 20 | (77) |
| *TGFB1* | SNP | missense | 19 | 41858876 | c.74G>C | p.(Arg25Pro) | rs1800471 | 4 | (11) | 3 | (12) |
| *TGFB1* | SNP | missense | 19 | 41847860 | c.788C>T | p.(Thr263Ile) | rs1800472 | 6 | (17) | 1 | (4) |
| *TGFB1* | SNP | missense | 19 | 41858864 | c.86G>C | p.(Gly29Ala) | rs199758510 | - | | 1 | (4) |
| *TNF* | SNP | missense | 6 | 31544333 | c.209T>C | p.(Ile70Thr) |  | 1 | (3) | - | |
| *TNF* | SNP | missense | 6 | 31543541 | c.23G>A | p.(Arg8Gln) | rs201502336 | 1 | (3) | - | |
| *TNFRSF11B* | SNP | splice_donor_+4 | 8 | 119945166 | c.400+4C>T |  | rs1564858 | 5 | (14) | 7 | (27) |
| *TNFRSF11B* | SNP | missense | 8 | 119964052 | c.9C>G | p.(Asn3Lys) | rs2073618 | 28 | (80) | 19 | (73) |
| *TNFRSF11B* | SNP | synonymous | 8 | 119938782 | c.768A>G | p.(Leu256=) | rs2228568 | 5 | (14) | 7 | (27) |
| *TNFRSF11B* | SNP | intronic | 8 | 119941173 | c.401-5T>C |  | rs3134046 | 35 | (100) | 26 | (100) |
| *VDR* | SNP | synonymous | 12 | 48249469 | c.699G>A | p.(Leu233=) | rs141863776 | 1 | (3) | - | |
| *VDR* | SNP | no-start | 12 | 48272895 | c.2T>C | p.(Met1?) | rs2228570 | 30 | (86) | 20 | (77) |
| *VDR* | SNP | missense | 12 | 48251360 | c.389G>A | p.(Arg130His) | rs371655308 | 1 | (3) | - | |
| *VDR* | SNP | synonymous | 12 | 48238757 | c.1056T>C | p.(Ile352=) | rs731236 | 16 | (46) | 15 | (58) |
| *VDR* | SNP | synonymous | 12 | 48258945 | c.162G>A | p.(Arg54=) | rs774173372 | 2 | (6) | - | |
| *VDR* | SNP | intronic | 12 | 48238837 | c.1025-49G>T |  | rs7975232 | 8 | (23) | 11 | (42) |
| *VDR* | SNP | synonymous | 12 | 48240209 | c.933G>A | p.(Glu311=) | rs150775215 | - | | 1 | (4) |
| *VDR* | SNP | synonymous | 12 | 48272840 | c.57C>T | p.(Asn19=) | rs2228572 | - | | 1 | (4) |
| *VDR* | SNP | missense | 12 | 48250920 | c.575C>G | p.(Thr192Ser) | rs540774973 | - | | 1 | (4) |

*p<0.05 compared to control group

**Supplementary Table S3. HLA-A, HLA-B, HLA-C, HLA-DQA1, HLA-DQB1 and HLA-DRB1 alleles identified in SLE patients (n=35) and controls (n=26).** Red color indicates HLA alleles found in greater numbers in SLE patients compared to controls. Purple color indicates a combination of HLA alleles found only in SLE patients.

| **SLE samples** | **Allele** | **HLA-A** | **HLA-B** | **HLA-C** | **HLA-DQA1** | **HLA-DQB1** | **HLA-DRB1** |
| --- | --- | --- | --- | --- | --- | --- | --- |
| **1** | ALLELE_1 | HLA-A*24:02 | HLA-B*35:01 | HLA-C*04:01 | HLA-DQA1*02:01 | HLA-DQB1*02:02 | HLA-DRB1*07:01 |
|  | ALLELE_2 | HLA-A*32:01 | HLA-B*35:03 | HLA-C*04:01 | HLA-DQA1*05:05 | HLA-DQB1*03:01 | HLA-DRB1*11:01 |
| **2** | ALLELE_1 | HLA-A*24:02 | HLA-B*35:03 | HLA-C*03:04 | HLA-DQA1*01:02 | HLA-DQB1*05:02 | HLA-DRB1*14:01 |
|  | ALLELE_2 | HLA-A*26:01 | HLA-B*50:01 | HLA-C*06:02 | HLA-DQA1*01:04 | HLA-DQB1*05:03 | HLA-DRB1*16:01 |
| **3** | ALLELE_1 | HLA-A*02:01 | HLA-B*51:01 | HLA-C*07:02 | HLA-DQA1*05:05 | HLA-DQB1*03:01 | HLA-DRB1*11:04 |
|  | ALLELE_2 | HLA-A*31:01 | HLA-B*51:01 | HLA-C*15:02 | HLA-DQA1*05:65 | HLA-DQB1*03:01 | HLA-DRB1*12:01 |
| **4** | ALLELE_1 | HLA-A*11:01 | HLA-B*07:02 | HLA-C*04:01 | HLA-DQA1*01:02 | HLA-DQB1*05:01 | HLA-DRB1*10:01 |
|  | ALLELE_2 | HLA-A*24:02 | HLA-B*35:01 | HLA-C*07:02 | HLA-DQA1*01:05 | HLA-DQB1*06:02 | HLA-DRB1*15:01 |
| **5** | ALLELE_1 | HLA-A*02:01 | HLA-B*13:02 | HLA-C*02:02 | HLA-DQA1*01:02 | HLA-DQB1*02:02 | HLA-DRB1*07:01 |
|  | ALLELE_2 | HLA-A*31:01 | HLA-B*27:02 | HLA-C*06:02 | HLA-DQA1*02:01 | HLA-DQB1*05:02 | HLA-DRB1*16:01 |
| **6** | ALLELE_1 | HLA-A*02:01 | HLA-B*35:01 | HLA-C*04:01 | HLA-DQA1*01:01 | HLA-DQB1*05:01 | HLA-DRB1*01:01 |
|  | ALLELE_2 | HLA-A*02:01 | HLA-B*51:01 | HLA-C*15:02 | HLA-DQA1*01:03 | HLA-DQB1*06:03 | HLA-DRB1*13:01 |
| **7** | ALLELE_1 | HLA-A*01:01 | HLA-B*27:05 | HLA-C*02:02 | HLA-DQA1*03:01 | HLA-DQB1*03:02 | HLA-DRB1*04:04 |
|  | ALLELE_2 | HLA-A*24:02 | HLA-B*37:01 | HLA-C*06:02 | HLA-DQA1*03:01 | HLA-DQB1*03:02 | HLA-DRB1*04:04 |
| **8** | ALLELE_1 | HLA-A*01:01 | HLA-B*08:01 | HLA-C*04:01 | HLA-DQA1*01:02 | HLA-DQB1*02:01 | HLA-DRB1*03:01 |
|  | ALLELE_2 | HLA-A*02:01 | HLA-B*35:01 | HLA-C*07:01 | HLA-DQA1*05:01 | HLA-DQB1*06:02 | HLA-DRB1*15:01 |
| **9** | ALLELE_1 | HLA-A*02:01 | HLA-B*13:02 | HLA-C*06:02 | HLA-DQA1*02:01 | HLA-DQB1*02:02 | HLA-DRB1*07:01 |
|  | ALLELE_2 | HLA-A*30:01 | HLA-B*18:01 | HLA-C*07:01 | HLA-DQA1*05:05 | HLA-DQB1*03:01 | HLA-DRB1*11:04 |
| **10** | ALLELE_1 | HLA-A*01:01 | HLA-B*18:01 | HLA-C*07:01 | HLA-DQA1*01:03 | HLA-DQB1*03:01 | HLA-DRB1*11:04 |
|  | ALLELE_2 | HLA-A*02:01 | HLA-B*52:01 | HLA-C*12:02 | HLA-DQA1*05:05 | HLA-DQB1*06:01 | HLA-DRB1*15:02 |
| **11** | ALLELE_1 | HLA-A*23:01 | HLA-B*35:08 | HLA-C*04:01 | HLA-DQA1*03:03 | HLA-DQB1*03:01 | HLA-DRB1*04:05 |
|  | ALLELE_2 | HLA-A*24:02 | HLA-B*49:01 | HLA-C*07:01 | HLA-DQA1*05:05 | HLA-DQB1*03:02 | HLA-DRB1*11:01 |
| **12** | ALLELE_1 | HLA-A*03:01 | HLA-B*49:01 | HLA-C*06:02 | HLA-DQA1*05:05 | HLA-DQB1*03:01 | HLA-DRB1*11:01 |
|  | ALLELE_2 | HLA-A*25:01 | HLA-B*50:01 | HLA-C*07:01 | HLA-DQA1*05:65 | HLA-DQB1*03:01 | HLA-DRB1*11:04 |
| **13** | ALLELE_1 | HLA-A*02:01 | HLA-B*49:01 | HLA-C*03:02 | HLA-DQA1*04:01 | HLA-DQB1*02:01 | HLA-DRB1*03:01 |
|  | ALLELE_2 | HLA-A*23:01 | HLA-B*58:01 | HLA-C*07:01 | HLA-DQA1*05:01 | HLA-DQB1*03:01 | HLA-DRB1*08:03 |
| **14** | ALLELE_1 | HLA-A*02:01 | HLA-B*13:02 | HLA-C*06:02 | HLA-DQA1*02:01 | HLA-DQB1*02:02 | HLA-DRB1*07:01 |
|  | ALLELE_2 | HLA-A*26:01 | HLA-B*14:01 | HLA-C*08:02 | HLA-DQA1*02:01 | HLA-DQB1*02:02 | HLA-DRB1*07:01 |
| **15** | ALLELE_1 | HLA-A*01:01 | HLA-B*40:02 | HLA-C*02:02 | HLA-DQA1*01:02 | HLA-DQB1*04:02 | HLA-DRB1*08:04 |
|  | ALLELE_2 | HLA-A*02:01 | HLA-B*58:01 | HLA-C*07:18 | HLA-DQA1*04:01 | HLA-DQB1*05:02 | HLA-DRB1*16:02 |
| **16** | ALLELE_1 | HLA-A*02:05 | HLA-B*44:46 | HLA-C*02:02 | HLA-DQA1*02:01 | HLA-DQB1*02:02 | HLA-DRB1*07:01 |
|  | ALLELE_2 | HLA-A*11:01 | HLA-B*50:01 | HLA-C*06:02 | HLA-DQA1*05:05 | HLA-DQB1*03:01 | HLA-DRB1*13:03 |
| **17** | ALLELE_1 | HLA-A*24:02 | HLA-B*35:01 | HLA-C*04:01 | HLA-DQA1*01:02 | HLA-DQB1*05:02 | HLA-DRB1*15:01 |
|  | ALLELE_2 | HLA-A*26:01 | HLA-B*52:01 | HLA-C*12:02 | HLA-DQA1*01:03 | HLA-DQB1*06:01 | HLA-DRB1*15:02 |
| **18** | ALLELE_1 | HLA-A*02:01 | HLA-B*07:02 | HLA-C*07:02 | HLA-DQA1*01:02 | HLA-DQB1*05:02 | HLA-DRB1*15:01 |
|  | ALLELE_2 | HLA-A*68:01 | HLA-B*51:01 | HLA-C*15:02 | HLA-DQA1*01:02 | HLA-DQB1*06:02 | HLA-DRB1*16:01 |
| **19** | ALLELE_1 | HLA-A*02:05 | HLA-B*50:01 | HLA-C*06:02 | HLA-DQA1*02:01 | HLA-DQB1*02:02 | HLA-DRB1*07:01 |
|  | ALLELE_2 | HLA-A*69:01 | HLA-B*50:01 | HLA-C*06:02 | HLA-DQA1*02:01 | HLA-DQB1*02:02 | HLA-DRB1*07:01 |
| **20** | ALLELE_1 | HLA-A*03:01 | HLA-B*35:02 | HLA-C*04:01 | HLA-DQA1*01:02 | HLA-DQB1*03:01 | HLA-DRB1*11:04 |
|  | ALLELE_2 | HLA-A*23:01 | HLA-B*38:01 | HLA-C*12:03 | HLA-DQA1*05:05 | HLA-DQB1*06:04 | HLA-DRB1*13:02 |
| **21** | ALLELE_1 | HLA-A*01:01 | HLA-B*18:01 | HLA-C*06:02 | HLA-DQA1*04:01 | HLA-DQB1*03:01 | HLA-DRB1*08:01 |
|  | ALLELE_2 | HLA-A*24:02 | HLA-B*37:01 | HLA-C*12:03 | HLA-DQA1*05:05 | HLA-DQB1*04:02 | HLA-DRB1*11:01 |
| **22** | ALLELE_1 | HLA-A*24:02 | HLA-B*18:01 | HLA-C*05:01 | HLA-DQA1*01:02 | HLA-DQB1*03:01 | HLA-DRB1*11:04 |
|  | ALLELE_2 | HLA-A*33:03 | HLA-B*44:29 | HLA-C*12:03 | HLA-DQA1*05:05 | HLA-DQB1*05:02 | HLA-DRB1*15:01 |
| **23** | ALLELE_1 | HLA-A*02:01 | HLA-B*14:02 | HLA-C*07:01 | HLA-DQA1*05:01 | HLA-DQB1*02:01 | HLA-DRB1*03:01 |
|  | ALLELE_2 | HLA-A*32:01 | HLA-B*18:01 | HLA-C*08:02 | HLA-DQA1*05:05 | HLA-DQB1*03:01 | HLA-DRB1*11:04 |
| **24** | ALLELE_1 | HLA-A*01:01 | HLA-B*35:01 | HLA-C*04:01 | HLA-DQA1*01:02 | HLA-DQB1*03:01 | HLA-DRB1*11:04 |
|  | ALLELE_2 | HLA-A*03:01 | HLA-B*44:27 | HLA-C*07:04 | HLA-DQA1*05:05 | HLA-DQB1*05:02 | HLA-DRB1*16:01 |
| **25** | ALLELE_1 | HLA-A*23:01 | HLA-B*35:01 | HLA-C*04:01 | HLA-DQA1*01:04 | HLA-DQB1*03:01 | HLA-DRB1*11:01 |
|  | ALLELE_2 | HLA-A*68:01 | HLA-B*44:02 | HLA-C*05:01 | HLA-DQA1*05:05 | HLA-DQB1*05:03 | HLA-DRB1*14:01 |
| **26** | ALLELE_1 | HLA-A*02:06 | HLA-B*15:01 | HLA-C*02:02 | HLA-DQA1*01:02 | HLA-DQB1*03:01 | HLA-DRB1*11:01 |
|  | ALLELE_2 | HLA-A*24:02 | HLA-B*27:02 | HLA-C*12:03 | HLA-DQA1*05:05 | HLA-DQB1*05:02 | HLA-DRB1*16:01 |
| **27** | ALLELE_1 | HLA-A*11:01 | HLA-B*37:01 | HLA-C*06:02 | HLA-DQA1*01:02 | HLA-DQB1*02:02 | HLA-DRB1*07:01 |
|  | ALLELE_2 | HLA-A*23:01 | HLA-B*49:01 | HLA-C*07:01 | HLA-DQA1*02:01 | HLA-DQB1*06:09 | HLA-DRB1*13:02 |
| **28** | ALLELE_1 | HLA-A*02:01 | HLA-B*07:02 | HLA-C*05:01 | HLA-DQA1*01:02 | HLA-DQB1*03:01 | HLA-DRB1*11:04 |
|  | ALLELE_2 | HLA-A*02:01 | HLA-B*51:01 | HLA-C*07:02 | HLA-DQA1*05:05 | HLA-DQB1*06:03 | HLA-DRB1*15:01 |
| **29** | ALLELE_1 | HLA-A*01:01 | HLA-B*18:01 | HLA-C*07:01 | HLA-DQA1*01:04 | HLA-DQB1*03:01 | HLA-DRB1*11:01 |
|  | ALLELE_2 | HLA-A*24:02 | HLA-B*18:01 | HLA-C*12:03 | HLA-DQA1*05:05 | HLA-DQB1*05:03 | HLA-DRB1*14:01 |
| **30** | ALLELE_1 | HLA-A*02:01 | HLA-B*35:01 | HLA-C*04:01 | HLA-DQA1*01:02 | HLA-DQB1*05:02 | HLA-DRB1*11:01 |
|  | ALLELE_2 | HLA-A*32:01 | HLA-B*35:08 | HLA-C*04:01 | HLA-DQA1*01:02 | HLA-DQB1*06:04 | HLA-DRB1*13:02 |
| **31** | ALLELE_1 | HLA-A*11:01 | HLA-B*18:01 | HLA-C*04:01 | HLA-DQA1*01:02 | HLA-DQB1*05:02 | HLA-DRB1*14:01 |
|  | ALLELE_2 | HLA-A*11:01 | HLA-B*35:01 | HLA-C*12:03 | HLA-DQA1*01:04 | HLA-DQB1*05:03 | HLA-DRB1*16:02 |
| **32** | ALLELE_1 | HLA-A*01:01 | HLA-B*35:01 | HLA-C*04:01 | HLA-DQA1*03:03 | HLA-DQB1*03:01 | HLA-DRB1*04:07 |
|  | ALLELE_2 | HLA-A*24:03 | HLA-B*51:01 | HLA-C*14:02 | HLA-DQA1*05:05 | HLA-DQB1*03:01 | HLA-DRB1*11:01 |
| **33** | ALLELE_1 | HLA-A*02:01 | HLA-B*51:01 | HLA-C*01:02 | HLA-DQA1*01:04 | HLA-DQB1*03:02 | HLA-DRB1*04:03 |
|  | ALLELE_2 | HLA-A*24:02 | HLA-B*51:01 | HLA-C*01:02 | HLA-DQA1*03:01 | HLA-DQB1*05:03 | HLA-DRB1*14:01 |
| **34** | ALLELE_1 | HLA-A*02:01 | HLA-B*15:08 | HLA-C*01:02 | HLA-DQA1*01:01 | HLA-DQB1*02:02 | HLA-DRB1*01:01 |
|  | ALLELE_2 | HLA-A*31:01 | HLA-B*56:01 | HLA-C*01:02 | HLA-DQA1*02:01 | HLA-DQB1*05:01 | HLA-DRB1*07:01 |
| **35** | ALLELE_1 | HLA-A*02:01 | HLA-B*39:01 | HLA-C*12:03 | HLA-DQA1*01:02 | HLA-DQB1*03:01 | HLA-DRB1*11:04 |
|  | ALLELE_2 | HLA-A*11:01 | HLA-B*51:01 | HLA-C*15:02 | HLA-DQA1*05:05 | HLA-DQB1*05:02 | HLA-DRB1*16:01 |

| **control samples** | **Allele** | **HLA-A** | **HLA-B** | **HLA-C** | **HLA-DQA1** | **HLA-DQB1** | **HLA-DRB1** |
| --- | --- | --- | --- | --- | --- | --- | --- |
| **1** | ALLELE_1 | HLA-A*02:01 | HLA-B*39:01 | HLA-C*03:03 | HLA-DQA1*01:02 | HLA-DQB1*05:02 | HLA-DRB1*16:01 |
|  | ALLELE_2 | HLA-A*11:01 | HLA-B*55:01 | HLA-C*12:03 | HLA-DQA1*01:02 | HLA-DQB1*05:02 | HLA-DRB1*16:01 |
| **2** | ALLELE_1 | HLA-A*01:01 | HLA-B*08:01 | HLA-C*06:02 | HLA-DQA1*02:01 | HLA-DQB1*02:01 | HLA-DRB1*03:01 |
|  | ALLELE_2 | HLA-A*02:01 | HLA-B*57:01 | HLA-C*07:01 | HLA-DQA1*05:01 | HLA-DQB1*03:03 | HLA-DRB1*07:01 |
| **3** | ALLELE_1 | HLA-A*02:01 | HLA-B*51:01 | HLA-C*14:02 | HLA-DQA1*01:01 | HLA-DQB1*05:01 | HLA-DRB1*01:01 |
|  | ALLELE_2 | HLA-A*24:02 | HLA-B*51:01 | HLA-C*16:02 | HLA-DQA1*01:03 | HLA-DQB1*06:03 | HLA-DRB1*13:01 |
| **4** | ALLELE_1 | HLA-A*02:01 | HLA-B*13:02 | HLA-C*05:01 | HLA-DQA1*01:01 | HLA-DQB1*02:02 | HLA-DRB1*01:01 |
|  | ALLELE_2 | HLA-A*30:01 | HLA-B*44:02 | HLA-C*06:02 | HLA-DQA1*02:01 | HLA-DQB1*05:01 | HLA-DRB1*07:01 |
| **5** | ALLELE_1 | HLA-A*02:01 | HLA-B*35:01 | HLA-C*02:02 | HLA-DQA1*01:01 | HLA-DQB1*05:01 | HLA-DRB1*16:02 or HLA-DRB1*01:01 |
|  | ALLELE_2 | HLA-A*11:01 | HLA-B*44:05 | HLA-C*04:01 | HLA-DQA1*01:02 | HLA-DQB1*05:02 | HLA-DRB1*16:02 or HLA-DRB1*01:01 |
| **6** | ALLELE_1 | HLA-A*01:01 | HLA-B*14:02 or HLA-B*44:02 | HLA-C*05:01 | HLA-DQA1*01:01 | HLA-DQB1*05:01 | HLA-DRB1*01:02 |
|  | ALLELE_2 | HLA-A*33:01 | HLA-B*14:02 or HLA-B*44:02 | HLA-C*08:02 | HLA-DQA1*01:04 | HLA-DQB1*05:03 | HLA-DRB1*14:01 |
| **7** | ALLELE_1 | HLA-A*03:01 | HLA-B*44:02 | HLA-C*05:01 | HLA-DQA1*01:03 | HLA-DQB1*03:01 | HLA-DRB1*11:04 |
|  | ALLELE_2 | HLA-A*24:02 | HLA-B*57:01 | HLA-C*06:02 | HLA-DQA1*05:05 | HLA-DQB1*06:03 | HLA-DRB1*13:01 |
| **8** | ALLELE_1 | HLA-A*02:01 | HLA-B*44:02 | HLA-C*05:01 | HLA-DQA1*02:01 | HLA-DQB1*03:01 | HLA-DRB1*07:01 |
|  | ALLELE_2 | HLA-A*02:01 | HLA-B*49:01 | HLA-C*07:01 | HLA-DQA1*05:05 | HLA-DQB1*03:03 | HLA-DRB1*12:01 |
| **9** | ALLELE_1 | HLA-A*02:01 | HLA-B*18:01 | HLA-C*04:01 | HLA-DQA1*01:02 | HLA-DQB1*03:01 | HLA-DRB1*11:01 |
|  | ALLELE_2 | HLA-A*11:01 | HLA-B*35:01 | HLA-C*12:03 | HLA-DQA1*05:05 | HLA-DQB1*06:02 | HLA-DRB1*15:01 |
| **10** | ALLELE_1 | HLA-A*02:17 | HLA-B*39:01 | HLA-C*12:03 | HLA-DQA1*01:02 | HLA-DQB1*03:03 | HLA-DRB1*09:01 |
|  | ALLELE_2 | HLA-A*26:01 | HLA-B*51:01 | HLA-C*15:02 | HLA-DQA1*03:02 | HLA-DQB1*05:02 | HLA-DRB1*16:01 |
| **11** | ALLELE_1 | HLA-A*03:01 | HLA-B*15:24 | HLA-C*03:03 | HLA-DQA1*03:03 | HLA-DQB1*03:01 | HLA-DRB1*04:10 |
|  | ALLELE_2 | HLA-A*26:01 | HLA-B*35:01 | HLA-C*03:03 | HLA-DQA1*05:05 | HLA-DQB1*04:02 | HLA-DRB1*11:04 |
| **12** | ALLELE_1 | HLA-A*01:01 | HLA-B*18:01 | HLA-C*05:01 | HLA-DQA1*05:01 | HLA-DQB1*02:01 | HLA-DRB1*03:01 |
|  | ALLELE_2 | HLA-A*01:01 | HLA-B*50:01 | HLA-C*06:02 | HLA-DQA1*05:01 | HLA-DQB1*02:01 | HLA-DRB1*03:01 |
| **13** | ALLELE_1 | HLA-A*26:01 | HLA-B*15:01 | HLA-C*03:03 | HLA-DQA1*03:01 | HLA-DQB1*03:02 | HLA-DRB1*04:01 |
|  | ALLELE_2 | HLA-A*31:01 | HLA-B*40:02 | HLA-C*03:03 | HLA-DQA1*04:01 | HLA-DQB1*04:02 | HLA-DRB1*08:02 |
| **14** | ALLELE_1 | HLA-A*11:01 | HLA-B*07:02 | HLA-C*07:02 | HLA-DQA1*01:02 | HLA-DQB1*05:04 | HLA-DRB1*01:01 |
|  | ALLELE_2 | HLA-A*24:03 | HLA-B*18:01 | HLA-C*12:03 | HLA-DQA1*01:02 | HLA-DQB1*06:03 | HLA-DRB1*15:01 |
| **15** | ALLELE_1 | HLA-A*02:01 | HLA-B*18:01 | HLA-C*06:02 | HLA-DQA1*01:02 | HLA-DQB1*03:01 | HLA-DRB1*11:01 |
|  | ALLELE_2 | HLA-A*03:01 | HLA-B*57:01 | HLA-C*07:01 | HLA-DQA1*05:05 | HLA-DQB1*05:02 | HLA-DRB1*16:01 |
| **16** | ALLELE_1 | HLA-A*03:01 | HLA-B*35:01 | HLA-C*02:02 | HLA-DQA1*03:03 | HLA-DQB1*03:01 | HLA-DRB1*04:01 |
|  | ALLELE_2 | HLA-A*69:01 | HLA-B*51:01 | HLA-C*04:01 | HLA-DQA1*05:05 | HLA-DQB1*03:01 | HLA-DRB1*11:01 |
| **17** | ALLELE_1 | HLA-A*03:01 | HLA-B*35:01 | HLA-C*04:01 | HLA-DQA1*01:01 | HLA-DQB1*05:01 | HLA-DRB1*01:01 |
|  | ALLELE_2 | HLA-A*11:01 | HLA-B*51:01 | HLA-C*16:02 | HLA-DQA1*01:03 | HLA-DQB1*06:03 | HLA-DRB1*13:01 |
| **18** | ALLELE_1 | HLA-A*24:02 | HLA-B*15:03 | HLA-C*02:10 | HLA-DQA1*01:02 | HLA-DQB1*05:01 | HLA-DRB1*10:01 |
|  | ALLELE_2 | HLA-A*32:01 | HLA-B*55:01 | HLA-C*03:07 | HLA-DQA1*01:05 | HLA-DQB1*05:02 | HLA-DRB1*16:01 |
| **19** | ALLELE_1 | HLA-A*02:01 | HLA-B*35:01 | HLA-C*02:02 | HLA-DQA1*01:01 | HLA-DQB1*03:01 | HLA-DRB1*01:01 |
|  | ALLELE_2 | HLA-A*11:01 | HLA-B*51:01 | HLA-C*04:01 | HLA-DQA1*05:05 | HLA-DQB1*05:01 | HLA-DRB1*11:01 |
| **20** | ALLELE_1 | HLA-A*26:01 | HLA-B*18:01 | HLA-C*12:03 | HLA-DQA1*01:02 | HLA-DQB1*03:01 | HLA-DRB1*11:01 |
|  | ALLELE_2 | HLA-A*32:01 | HLA-B*39:01 | HLA-C*12:03 | HLA-DQA1*05:05 | HLA-DQB1*05:02 | HLA-DRB1*16:01 |
| **21** | ALLELE_1 | HLA-A*01:01 | HLA-B*08:01 | HLA-C*07:01 | HLA-DQA1*05:01 | HLA-DQB1*02:01 | HLA-DRB1*03:01 |
|  | ALLELE_2 | HLA-A*02:01 | HLA-B*18:01 | HLA-C*07:02 | HLA-DQA1*05:05 | HLA-DQB1*03:01 | HLA-DRB1*11:04 |
| **22** | ALLELE_1 | HLA-A*02:01 | HLA-B*15:01 | HLA-C*03:03 | HLA-DQA1*01:02 | HLA-DQB1*03:02 | HLA-DRB1*04:01 |
|  | ALLELE_2 | HLA-A*03:01 | HLA-B*44:02 | HLA-C*07:04 | HLA-DQA1*03:01 | HLA-DQB1*05:02 | HLA-DRB1*16:01 |
| **13** | ALLELE_1 | HLA-A*01:01 | HLA-B*18:01 | HLA-C*07:01 | HLA-DQA1*01:03 | HLA-DQB1*03:01 | HLA-DRB1*11:04 |
|  | ALLELE_2 | HLA-A*02:01 | HLA-B*52:01 | HLA-C*12:02 | HLA-DQA1*05:05 | HLA-DQB1*06:01 | HLA-DRB1*15:02 |
| **24** | ALLELE_1 | HLA-A*11:01 | HLA-B*35:01 | HLA-C*04:01 | HLA-DQA1*03:01 | HLA-DQB1*02:01 | HLA-DRB1*03:01 |
|  | ALLELE_2 | HLA-A*30:04 | HLA-B*41:01 | HLA-C*16:02 | HLA-DQA1*05:01 | HLA-DQB1*03:02 | HLA-DRB1*04:02 |
| **25** | ALLELE_1 | HLA-A*02:01 | HLA-B*13:02 | HLA-C*04:01 | HLA-DQA1*02:01 | HLA-DQB1*02:02 | HLA-DRB1*07:01 |
|  | ALLELE_2 | HLA-A*11:01 | HLA-B*53:01 | HLA-C*06:02 | HLA-DQA1*05:05 | HLA-DQB1*03:01 | HLA-DRB1*11:01 |
| **26** | ALLELE_1 | HLA-A*01:01 | HLA-B*51:01 | HLA-C*01:02 | HLA-DQA1*04:01 | HLA-DQB1*03:01 | HLA-DRB1*08:01 |
|  | ALLELE_2 | HLA-A*32:01 | HLA-B*51:01 | HLA-C*16:02 | HLA-DQA1*05:03 | HLA-DQB1*04:02 | HLA-DRB1*12:01 |
